# Inflammatory mechanisms contribute to long-term cognitive deficits induced by perinatal asphyxia via interleukin-1

**DOI:** 10.1038/s41386-025-02227-8

**Published:** 2025-10-02

**Authors:** Hanga Kelemen, Gyula Y. Balla, Kornél Demeter, Eszter Sipos, András Buzás-Kaizler, László Biró, Manó Aliczki, Barbara Orsolits, Áron Kerényi, Zoltán Balogh, Benedek Pászthy-Szabó, Diána Pejtsik, Lajos Hegyi, Krisztián Szigeti, Domokos Máthé, Parasuraman Padmanabhan, Csaba Bödör, Andrea Fekete, Miklós Szabó, Kai Kaila, Ádám Dénes, Éva Mikics

**Affiliations:** 1 Translational Behavioural Neuroscience Research Group, HUN-REN Institute of Experimental Medicine, Budapest, Hungary; 2 János Szentágothai Neurosciences Division, Doctoral College, Semmelweis University, Budapest, Hungary; 3 Behavioural Studies Unit, HUN-REN Institute of Experimental Medicine, Budapest, Hungary; 4 Laboratory of Cellular Neuropharmacology, HUN-REN Institute of Experimental Medicine, Budapest, Hungary; 5 “Momentum” Laboratory of Neuroimmunology, HUN-REN Institute of Experimental Medicine, Budapest, Hungary; 6 HCEMM-SU Molecular Oncohematology Research Group, Department of Pathology and Experimental Cancer Research, Semmelweis University, Budapest, Hungary; 7 MTA-SE Lendület Molecular Oncohematology Research Group, Department of Pathology and Experimental Cancer Research, Semmelweis University, Budapest, Hungary; 8 Department of Biophysics and Radiation Biology, Semmelweis University, Budapest, Hungary; 9 HCEMM-SU In Vivo Imaging Advanced Core Facility, Semmelweis University, Budapest, Hungary; 10 Cognitive Neuroimaging Centre, Nanyang Technological University, Singapore, Singapore; 11 Pediatric Center, MTA Center of Excellence, Semmelweis University, Budapest, Hungary; 12 MTA-SE Lendület “Momentum” Diabetes Research Group, Semmelweis University, Budapest, Hungary; 13 Department of Neonatology, Pediatric Center, MTA Center of Excellence, Semmelweis University, Budapest, Hungary; 14 Neuroscience Center, University of Helsinki, Helsinki, Finland

**Keywords:** Neuroscience, Psychiatric disorders

## Abstract

Perinatal asphyxia remains a leading cause of neonatal mortality and a major contributor to permanent neurological deficits. Even mild cases can result in long-term neurodevelopmental, cognitive, behavioural and psychiatric disorders. However, the mechanisms underlying asphyxia-induced hypoxic-ischaemic brain injury remain poorly understood, limiting the development of targeted interventions during the critical early plastic period. To explore the behavioural and molecular outcomes of perinatal asphyxia that may model important aspects of neuropsychiatric disorders observed in humans, we utilised a translationally relevant, non-invasive oxygen deprivation model of asphyxia in postnatal day 7 rats. We conducted a comprehensive assessment of asphyxia-induced changes, integrating neurobehavioural profiling (evaluating cognitive, emotional, social and neuromotor functions), microglial morphology analysis, neuroimaging, stress hormone measurement and whole-transcriptome sequencing techniques to elucidate the acute and long-term functional consequences. Consistent with clinical observations, the extensive functional assessment revealed distinct sex-dependent effects, including increased anxiety and impulsivity, attention deficits and impaired inhibitory control, which were observed exclusively in males, with no apparent sensorimotor deficits. This phenotype resembling attention deficit hyperactivity disorder (ADHD) in adult rats was associated with a lasting increase in inhibitory bouton densities in the medial prefrontal cortex. The development of an acute inflammatory response after perinatal asphyxia marked by phenotypic transformation of microglia, paralleled brain perfusion and stress hormone changes. Notably, microglial changes were mitigated by the blockade of proinflammatory interleukin-1 signalling via systemic IL-1 receptor antagonist (IL-1RA) administration in a therapeutically relevant time window. Importantly, early blockade of proinflammatory responses was able to prevent cognitive deficits in adulthood and normalise inhibitory bouton densities. RNA sequencing analysis revealed asphyxia-induced dysregulation of molecular pathways targeting GABAergic signalling, potentially contributing to subsequent morphological and neuropsychiatric alterations. IL-1RA treatment appeared to engage distinct epigenetic regulatory mechanisms, rather than merely reversing these disruptions in the acute post-asphyxia period. Collectively, these findings demonstrate that perinatal asphyxia induces marked behavioural deficits in attention and inhibitory control, paralleled by lasting inhibitory and epigenetic dysregulation, preceded by acute induction of microglia-driven inflammatory processes in the medial prefrontal cortex. Systemic IL-1RA administration may represent a promising therapeutic opportunity to prevent long-term cognitive impairments caused by perinatal asphyxia.

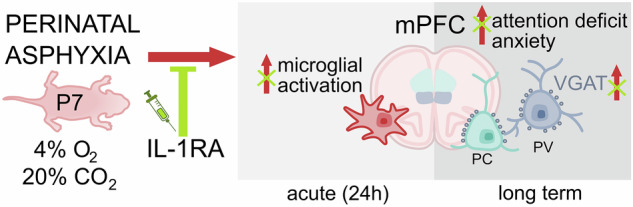

## Introduction

To date, perinatal asphyxia (PA) remains a substantial burden on healthcare systems worldwide and a leading cause of child mortality, accounting for approximately 23% of all newborn deaths globally (World Health Organisation, 2022). Among infants who survive PA, roughly 25% develop permanent neurological disabilities, ranging from cerebral palsy, epilepsy and cerebral visual impairment to a spectrum of neuropsychiatric disorders, while potentially sustaining multi-organ injury beyond the brain [1, 2]. Even mild to moderate PA, without significant sensorimotor sequelae, is linked to neurodevelopmental disorders, including attention-deficit hyperactivity disorder (ADHD) [3, 4], autism spectrum disorder [5], cognitive and communication deficits [2, 6–8], or stress-related psychiatric diagnoses, such as anxiety or affective disorders [9, 10].

Despite the enormous public health problem caused by PA and the extensive preclinical efforts to understand disease mechanisms, whole-body hypothermia remains the primary, most efficient, nonetheless a broadly non-specific therapeutic approach for moderate to severe birth asphyxia [11–13]. However, studies suggest that despite therapeutic cooling, a significant proportion of surviving neonates -estimated at approximately 30%- may still develop long-term neuropsychiatric deficits [12, 14]. Furthermore, the effectiveness of hypothermia treatment in low- and middle-income countries remains under discussion [13, 15, 16]. Inflammation is increasingly recognised as a key contributor to PA-induced brain injury, affecting neuronal circuits, glial cells and cerebral perfusion [17–19]. In line with this, clinical and preclinical studies confirm the functional role of inflammation in the development of cognitive impairments in several neuropsychiatric conditions, including neurodevelopmental disorders [20–24].

Early life stress paradigms, known to activate the hypothalamic-pituitary axis (HPA axis), have been demonstrated to trigger lasting changes in microglia functioning, affecting their density and phenotype (soma size, length and thickness of processes), and inflammatory responses, thereby disrupting their developmental programmes [25]. Moreover, inflammatory mediators and altered glial cell phenotypes have been implicated in shaping the trajectory of injury in brain regions responsible for higher-order cognitive processing via interactions with synaptic elements, which are particularly vulnerable to neonatal hypoxic-ischaemic damage [26–31]. Phenotypic transformation involving an increase in soma size and reduction of branching of microglial cells and related production of proinflammatory mediators such as interleukin-1 (IL-1) are well-established contributors to oxidative stress and neural damage [32–35]. Elevated levels of IL-1 have been strongly associated with the severity of PA [36]. Notably, microglial dysfunction and IL-1 actions markedly alter cerebral perfusion under both physiological conditions and after ischaemia [37, 38]. Nevertheless, the causal mechanisms by which these neuroimmunological interactions mediate long-term cognitive dysfunction in mild to moderate PA remain to be elucidated.

The medial prefrontal cortex (mPFC) is an essential region for top-down control of decision-making and behavioural regulation, playing a significant role in attention and emotional functioning [39, 40]. Dysfunction in the mPFC has been implicated in various neurodevelopmental disorders [41, 42], and has been linked to the effects of early-life adversities [43, 44]. Hence, this study focused on the functional, histological and molecular alterations in the mPFC induced by PA.

The main goal of this study was to investigate the putative neuroinflammatory mechanisms through which PA may contribute to the subsequent behavioural and histological alterations observed in adulthood, with an outlook on acute cerebral perfusion and gene expression. To this end, we utilised a translationally relevant non-invasive rodent model of mild to moderate PA, which enabled us to decipher the role of microglial activation in influencing disease progression via acute pharmacological intervention. This approach aimed to provide important insights into the pathophysiology of PA-induced neuropsychiatric impairments and identify potential therapeutic targets for mitigating the long-term consequences of PA.

## Materials and methods

### Animals

Experiments followed the European Communities Council Directive (2010/63/EU), the Council on Animal Care of the National Health Institution of Hungary (PEI/001/828-4/2015) guidelines and were approved by the Animal Welfare Committee of the HUN-REN Institute of Experimental Medicine (HUN-REN IEM, Budapest, Hungary). Male and female Wistar rats (Charles River Laboratories, Germany) from the HUN-REN IEM breeding colony were used. Details on housing, breeding and pup handling are provided in the Supplementary materials and methods.

### Perinatal asphyxia insult (PA)

The approach for the PA insult was based on the seminal work of Pospelov et al. [45], which established key methodological and physiological foundations for this model by inducing pathophysiological alterations in acid-base balance during PA [46–48] that closely resemble those observed in human neonates. The model was further modified to suit our study purposes. At P7, male and female pups were placed in isothermal treatment chambers and received a PA-inducing gas mixture (4% O_2_ and 20% CO_2_ in N_2_) for 15 min. Details about the PA induction are provided in the Supplementary materials and methods.

### Behavioural testing

Several cohorts of animals were used for behavioural assessment to avoid over-testing and minimise potential inter-test effects. The overall structure of behavioural testing and the specific tests assigned to each cohort are provided in a Supplementary Table 4. Animals were tested on P8 for acute severity scoring and at juvenile and adult ages for long-lasting effects. Detailed description of the behavioural tests appears in Supplementary materials and methods.

### Immunohistochemical studies

To examine the histological substrates of the phenotypic modifications, a subset of P8 and adult male animals was anaesthetised and transcardially perfused, and immunohistochemical staining was performed on mPFC slices. Detailed description of fixation, tissue processing, fluorescent immunostaining, synaptic puncta and microglial morphology analysis appears in Supplementary materials and methods.

### Acute hormone measurements

For the estimation of acute hormonal changes caused by PA, trunk blood was collected from male pups at P7 in baseline conditions (less than 5 min after separation from dam) and after PA (0 h, 1 h, 4 h and 24 h post-PA). ACTH, corticosterone and aldosterone concentrations were determined by radioimmunoassay (RIA), measuring all samples in the same assay. Blood sampling, hormone measurements and analysis are detailed in Supplementary materials and methods.

### SPECT and MRI imaging

The effect of PA on cerebral blood volume was assessed 24 h post-PA by dextran-coated iron oxide nanoparticles determined by 1 Tesla MRI volumetry [49]. Brain perfusion was measured by Single-Photon Emission CT (SPECT) imaging with 99mTc-HMPAO (Hexamethylpropyleneamine Oxime; Medi-Radiopharma Ltd., Budapest, Hungary). For methodological details, see Supplementary materials and methods.

### Pharmacological treatment

To assess and influence inflammation-related mechanisms in the transmission of PA-caused effects, male animals were treated with subcutaneous IL-1 receptor antagonist (IL-1RA, Kineret, Swedish Orphan Biovitrum AB) at a dose of 100 mg/kg. A volume of 10 μl/g IL-1RA dissolved in sterile 0.1% bovine serum albumin (BSA) was diluted in phosphate-buffered saline (PBS) and administered at 1 and 20 h after the PA insult to influence the primary and secondary phase of PA-induced energy failure [50].

### RNA sequencing and analysis

To decipher the acute gene expression changes caused by PA, brains of P8 and adult (6 months old) male animals were harvested under deep anaesthesia, and bilateral medial prefrontal cortices were microdissected (*N* = 8–10/treatment group). RNA isolation, library preparation and analysis are detailed in the Supplementary materials and methods.

### Statistical analysis

All measurements and analyses were performed in a blinded manner, following the STAIR and ARRIVE guidelines. For details on blinding, sample size calculation and outlier exclusion, see Supplementary materials and methods and Supplementary Tables 7 and 8. Behavioural data are presented as mean ± SEM. Behavioural outcomes of male and female treatment groups were analysed separately. Statistical analyses were performed using GraphPad Prism 8.0.1, Python 3.10 and R 4.2 (microglia analysis). Normality and variance assumptions were assessed using Shapiro-Wilk and Brown-Forsythe tests (Supplementary Table 5). Two-tailed unpaired *t*-tests were used when assumptions were met (t(df); p). Otherwise, Mann-Whitney *U* tests were applied (U; p). For four-group comparisons, two-way ANOVA with Tukey’s or Duncan’s post hoc tests was used. Repeated measures ANOVA was used for multi-day behavioural testing. Behavioural tests involving repeated measures were evaluated for the assumption of sphericity. Where violations were detected, corrections were applied using Greenhouse-Geiser (GG) and Huynh-Feld (HF) estimates (Supplementary Table 6). Degrees of freedom and exact *p* values are reported; α was set at 0.05.

## Results

### PA leads to long-term affective and cognitive dysfunction in males

**Fig. 1:**
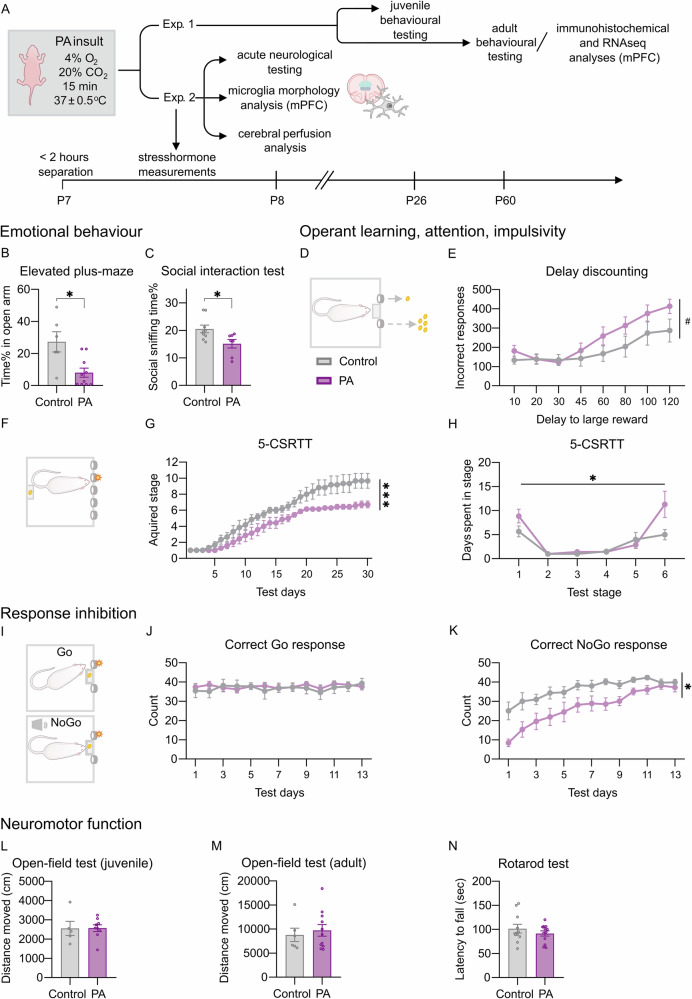
Long-term behavioural consequences of the PA insult in males. **A** Experimental design and PA insult. Seven-day-old male Wistar rat pups were subjected to 15 min of exposure to an asphyxia-inducing gas mixture (4% O_2_, 20% CO_2_) under normothermic conditions. In Experiment 1, different cohorts of animals underwent comprehensive behavioural testing from their juvenile period through adulthood, followed by immunohistochemical or differential gene expression analysis of the prefrontal cortex, likely to be involved in the observed deficits. In Experiment 2, animals were extensively characterised in the acute post-PA period (see Fig. 3). **B** Anxiety testing in the elevated plus-maze performed in young adulthood revealed a significant decrease in time spent in the open arms in the PA group (*U* = 9, *p* = 0.022). **C** PA animals showed slightly decreased social sniffing time in the social interaction test (*U* = 11, *p* = 0.031). **D**, **E** In the delay discounting task, PA animals exhibited marked motor impulsivity, indicated by the progressive increase in inadequate responses with the lengthening delay (the slope of the number of inadequate responses: *t* = 2.078, *p* = 0.055; insult × delay interaction *F*(7, 105) = 1.870, *p* = 0.081). **F**–**H** PA animals presented learning and attention deficits in the 5-choice serial reaction time task (acquired stage: test day *F*(2.015, 22.17) = 72.73, *p* < 0.001; treatment *F*(1,11) = 9.390, *p* = 0.010; test day × treatment interaction *F*(29,319) = 2.358, *p* < 0.001. PA animals were significantly slower in acquiring the task and were less able to proceed to more difficult stages (stage F (1.801,19.82) = 14,34, *p* < 0.001; treatment *F*(1,11) = 7.421, *p* = 0.019; stage × treatment interaction *F*(5,55) = 2.716, *p* = 0.029). This was the most prominent in stages 1 and 6. **I**–**K** In the Go/no-Go task, although PA animals were able to learn the Go cue (**J**), they showed marked inhibitory deficits when presented with the no-Go stimulus (**K**; trial day *F*(2.914,37.88) = 20.67, *p* < 0.001; treatment *F*(1,13) = 6.535, *p* = 0.023; trial day × treatment interaction *F*(12,156) = 2.028, *p* = 0.025). No significant differences in gross neuro-motor functioning were apparent between treatment groups during juvenile (**L**) and long-term assessment (**M**, **N**). Animals are depicted as individual data points. Error bars represent mean ± SEM; *p < 0.05; **p < 0.01; *** *p* < 0.001.

To investigate long-term functional consequences of PA, rats underwent a comprehensive behavioural assessment during their juvenile period or adulthood, first focusing on emotional, social and cognitive domains. Adult PA animals exhibited higher anxiety, revealed by reduced open arm exploration in the EPM (Fig. S1B; Fig. 1B). Social affective functioning was also influenced by PA, as suggested by decreased social sniffing during the social interaction test (Fig. 1C). No differences were observed in play behaviour during the play-fight test, sociability index in the sociability test and territorial aggression in the resident-intruder test (Fig. S1D–H).

Functional testing in automated operant systems revealed substantial and specific deviances in the behaviour of PA animals during both juvenile and adult periods. In the delay discounting task, a sensitive test for the quantification of impulsive behaviour in rodents [51], although both groups reached similar large reward preference during training (Fig. S1I), PA animals showed a strong tendency for enhanced motor impulsivity (Fig. 1D, E). Furthermore, PA resulted in significant attention deficits in the 5-CSRTT in adulthood. PA animals exhibited learning and attention impairments, progressing more slowly through stages requiring higher attentional performance. These deficits were particularly evident in stages demanding task comprehension (stage 1) or requiring shorter stimulus duration with fewer omissions (stage 6; Fig. 1F–H).

These behavioural findings of attention deficit, together with increased impulsivity, imply an ADHD-like phenotypic shift in PA animals, likely involving the mPFC [52]. To further investigate this assumption, we performed the Go/no-Go task in adulthood (modified for rodent testing; Fig. 1I), in which clinically diagnosed ADHD patients often perform poorly [53]. Both treatment groups executed the Go task requiring operant learning skills similarly (Fig. S 1J; Fig. 1J). PA rats, however, presented a significant decrease in correct no-Go responses during the no-Go phase, indicating impaired inhibition of a previously learned operant response (Fig. 1K). This suggests a deficit in withholding a prepotent response, a core feature of ADHD-like behaviour. These findings suggest a possible PA-related mPFC impairment, which specifically targets behavioural response inhibition.

The absence of late-onset gross motor function and coordination deficits was confirmed by similar locomotion in juvenile and adult OF tests and EPM (Fig. 1L, M; Fig. S1C) and latency to fall in the accelerating rotarod test (Fig. 1N). These data underpin our model’s suitability for studying asphyxia-related psychiatric consequences, as motor deficits would potentially mask fine-tuned emotional and cognitive disturbances.

We found no significant difference in working memory in the Y-maze test at a juvenile age (Fig. S1K). PA animals displayed decreased spatial learning and memory, as demonstrated by lower correct response percentages in the hole-board test (Fig. S1L). Adult PA animals showed impaired spatial learning in the Morris Water Maze, taking significantly longer to find the hidden platform over the trials (Fig. S1M).

To investigate the sex-dependency of the observed behavioural alterations, female animals underwent targeted assessment. As early-life adversities have been shown to affect emotional regulation in females [54, 55], we also investigated associative emotional learning in the conditioned fear learning paradigm. Female PA animals did not exhibit motor alterations in the OF (Fig. S2A). No differences were found in anxiety-like behaviour, fear learning and acquisition and sociability between control and PA females (Fig. S2B–E). Female PA animals showed no differences in learning, memory or impulsivity during the set-shifted 5-CSRTT when compared to female controls (Fig. S2F–H). Although females may also exhibit molecular alterations, we focused downstream analyses on males, as they showed the most robust behavioural phenotype following PA, providing a stronger basis for investigating the underlying molecular mechanisms.

### Long-term alteration of inhibitory synaptic markers in the medial prefrontal cortex after PA in males

Given the established role of the mPFC in attention, memory and behavioural inhibition by literature [39, 56–58], the behavioural deficits observed in PA males in the current study may reflect impairments in mPFC-dependent functions. Therefore, we investigated potential molecular alterations in the basic signalling apparatus within this region in adults. Confocal imaging analysis demonstrated no changes in neuronal numbers, indicated by the density of NeuN(+) cells (Fig. S3A–C). However, there was a notable increase in MBP(+) myelin basic protein density in the IL (Fig. S3J–L). No quantitative differences were apparent in Iba1(+) microglia and GFAP(+) astrocytes (Fig. S3D–I).

**Fig. 2:**
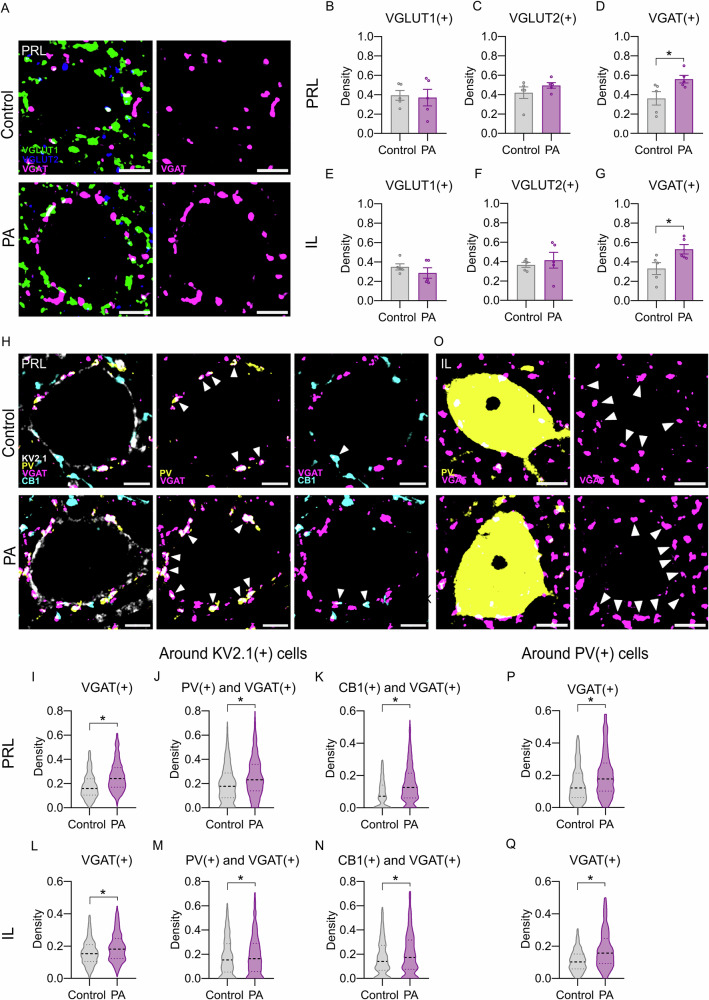
Confocal imaging reveals long-term alterations in mPFC inhibitory functioning after PA in males. **A**–**G** Long-term immunohistochemical characterisation of medial prefrontal cortical synaptic boutons in PA males (Exp.1). A marked increase in the density of VGA*T*(+) inhibitory terminals was observed in both the prelimbic (**D**; *t*(8) = 2.483, *p* = 0.037) and infralimbic (**G**; *t*(8) = 2.542, *p* = 0.034) subregions of the mPFC in PA-exposed adults. In contrast, there were no changes in the bouton density of VGLUT1(+) and VGLUT2(+) excitatory terminals (**B**, **C**; **E**, **F**). **H**–**N** Cell-type specific analysis of perisomatic boutons revealed a significant increase of VGA*T*(+) boutons in the closeness of KV2.1(+) pyramidal somas in both PRL (**I**; *U* = 190222, *p* < 0.001) and IL (**L**; *U* = 219272, *p* < 0.001). Colocalization analysis of VGAT and PV(+) or CB1(+) boutons revealed an increase in both bouton types in the PRL (**J**: PV(+) and VGA*T*( + ): *U* = 230013, *p* < 0.001; **K**: CB1(+) and VGA*T*(+): U = 291813, *p* < 0.001), but only CB1 in IL (**N**: VGA*T*(+) around KV2.1 U = 219272, *p* < 0.01; CB1(+) and VGA*T*(+): *U* = 295865, *p* < 0.001). **P**, **Q** The density of inhibitory boutons around PV(+) interneurons was also significantly increased in both regions (PRL *U* = 99215, *p* < 0.001; IL *U* = 50847, *p* < 0.001). Animals are depicted as individual data points in (**B**–**G**). Data are shown as normalised bouton densities across experimental groups with median and quartiles on (**I**–**Q**). Error bars represent mean ± SEM; *p < 0.05.

To further assess the molecular machinery underlying excitatory and inhibitory neurotransmission that may be persistently affected by PA [59], we quantified inhibitory (VGAT-positive) and excitatory (VGLUT1- and VGLUT2-positive) boutons in the mPFC. Confocal image analysis revealed a profound increase in VGAT(+) terminal density in the infralimbic (IL) and prelimbic (PRL) subregions in PA animals. In contrast, no changes were observed in VGLUT1(+) and VGLUT2(+) excitatory bouton densities (Fig. 2A–G). Perisomatic VGAT(+) boutons demonstrated an increased density surrounding KV2.1(+) pyramidal cells in the PRL and IL (Fig. 2H, I, L). Colocalization analysis revealed elevation in VGAT boutons associated with PV- and CB1-positive puncta around pyramidal cells (Fig. 2J, K), indicating an increase in both interneuron types in the close surroundings of PRL pyramidal cells. In contrast, the IL region showed an increase only in CB1(+) perisomatic inhibitory synapses around KV2.1(+) pyramidal cells (Fig. 2M, N). Notably, an increase in inhibitory bouton density neighbouring PV(+) interneurons was observed in both PRL and IL (Fig. 2O–Q).

To further elucidate the long-term molecular changes following PA, we conducted RNA sequencing on mPFC samples from adult PA and control males under baseline conditions. While gene-level differences did not reach statistical significance, gene set enrichment revealed upregulation of pathways related to epigenetic regulation and immune responses, and downregulation of transport and signalling pathways (Fig. S6A, B). Hierarchical clustering of altered pathways highlighted key domains involving histone methylation, immune function, cell signalling and membrane/synaptic transmission (Fig. S6C).

### PA induces an acute stress hormone surge and mPFC microglia phenotype alterations, without neurological deficits

**Fig. 3:**
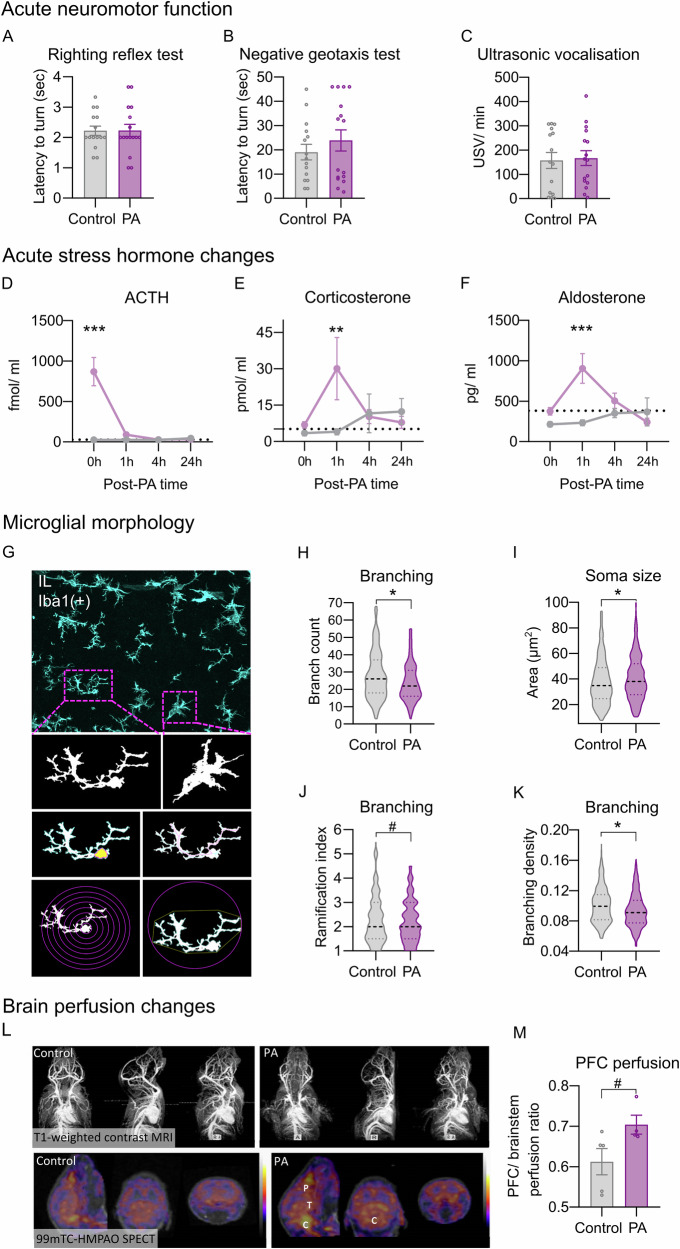
Acute effects of the PA insult in males. **A**–**C** Characterisation of the acute post-PA period, focusing on neuromotor functioning, stress hormone levels and regional microglial morphological changes (Exp. 2). No significant alterations in gross neuromotor functioning and the frequency of USV vocalisation were observed 24 h after PA. **D**–**F** PA leads to an acute burst of peripheral stress-related hormone levels in the immediate post-PA period. ACTH: treatment *F*(1, 48) = 19.26, *p* < 0.001; sampling time *F*(1, 48) = 16.6, *p* < 0.001; treatment × sampling time interaction *F*(3, 48) = 17.12, *p* < 0.001; Duncan’s post-hoc test for 0 h *p* < 0.001; corticosterone: treatment *F*(1, 48) = 2.714, *p* = 0.105; sampling time *F*(1, 48) = 2.151, *p* = 0.106; treatment × sampling time interaction *F*(3, 48) = 3.111, *p* = 0.034; Duncan’s post-hoc test for 1 h *p* = 0.006; aldosterone: treatment *F*(1, 35) = 10.815, *p* = 0.002; sampling time *F*(1, 35) = 3.643, *p* = 0.021; treatment × sampling time interaction *F*(1, 35) = 5.561, *p* = 0.003; Duncan’s post-hoc test for 1 h *p* < 0.001. **G** Representative confocal microscopy images of Iba1-positive microglia in the IL show markedly decreased microglia branch count (**H**; *U* = 62163, *p* < 0.001), ramification index (**J**; calculated from the ratio of max intersections and the number of primary branches; *U* = 68015, *p* = 0.068) and branch density (**K**; U = 63589, *p* < 0.001), and a tendency for increased soma size (**I**; U = 67747, *p* = 0.065). Data shown represent microglial cells across experimental groups with median and quartile. **L** Representative images of T1-weighted dextran-coated iron oxide contrast MRI (top) and 99mTC-HMPAO SPECT (bottom) neuroimaging (P = PFC, T = Thalamus, C = Cerebellum) demonstrate regional cerebral blood flow and perfusion changes. The colour scale corresponds to the relative intensity of radiotracer accumulation, with warmer colours indicating higher, while cooler colours indicating lower uptake. **M** SPECT imaging shows strong tendencies in the prefrontal cortex compared to the brainstem of PA animals (*U* = 2, *p* = 0.063). Error bars represent mean ± SEM; ***p < 0.001; *p < 0.05; #*p* < 0.07.

To explore the potential acute functional alterations that may contribute to the observed long-term behavioural and histological changes, we conducted a comprehensive characterisation of the first 24 h following PA in males, focusing on behaviour, stress hormone levels, histological and brain-perfusion alterations. Neurodevelopmental reflex tests [60] did not reveal group differences in the righting reflex test, negative geotaxis test or in ultrasonic vocalisation, indicating no early-onset robust neurological deficits (Fig. 3A–C; Fig. S1A).

Stress hormone disruptions were evident acutely after PA. A robust increase in blood ACTH levels was observed immediately after the insult (Fig. 3D), followed by a rise in corticosterone (Fig. 3E) and aldosterone levels (Fig. 3F) at 1-h post-PA. Intriguingly, both groups exhibited low corticosterone levels, reflecting reduced secretory capacity characterising the neonatal stress-hyporesponsive period (SHRP).

As an acutely emerging local and systemic inflammatory response plays a crucial role in the development of cognitive impairments in various hypoxic-ischaemic brain injuries, morphological analysis of resident microglia was performed 24 h after PA in the IL (Fig. 3G). PA led to acute microglial morphological transformation, indicated by significantly decreased branch count (Fig. 3H) and branch ramification (branching density; ramification index; Fig. 3J, K) and tendentious increase in soma size (Fig. 3I). Treatment groups presented similar numbers of microglia cell density (Fig. S4A, B).

### SPECT and MR imaging reveal subtle acute perfusion changes caused by PA

For the quantitative assessment of central nervous system (CNS) perfusion changes 24 h after PA, regional cerebral blood flow (rCBF) and whole-brain blood volume were measured in males via SPECT imaging using 99mTC-HMPAO and T1-weighted MRI, respectively (Fig. 3L). No differences in overall cerebral blood volume were identified (Fig. S4C). However, SPECT imaging revealed a tendency towards increased relative perfusion in the PFC (Fig. 3M) and thalamus (Fig. S4E) and a significant increase in the cerebellum (Fig. S4F) in PA animals. These findings suggest the presence of a relative post-insult increase in regional perfusion and subtle circulatory disturbances acutely after PA.

### IL-1RA ameliorates long-term deficits and influences microglia morphology changes in PA males

**Fig. 4:**
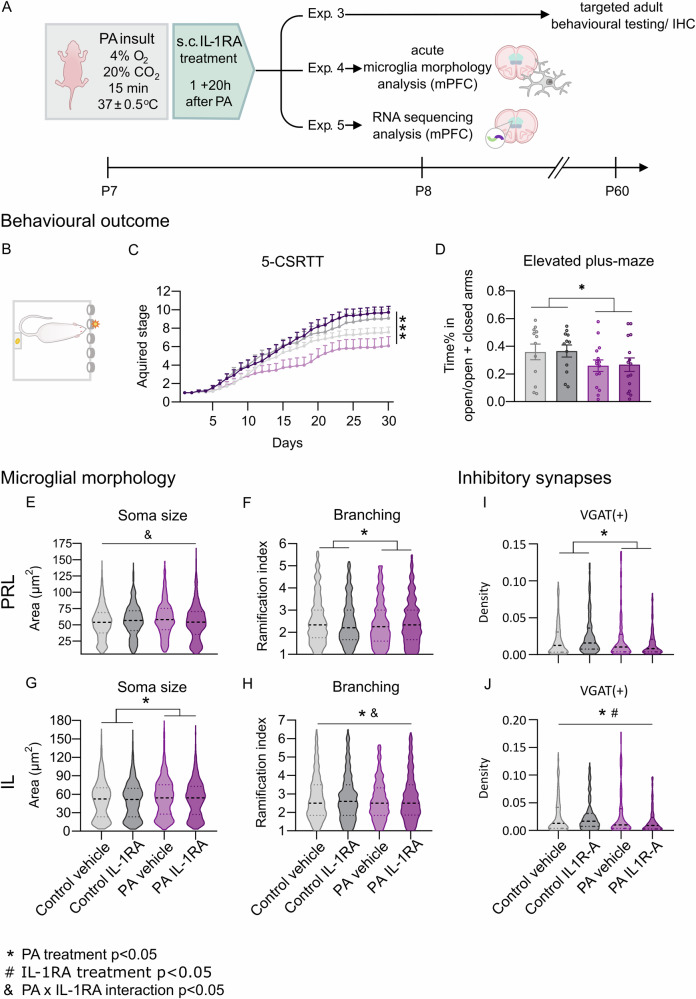
IL-1RA treatment prevents long-term attention deficits and shapes mPFC microglia morphology following PA. **A** Experimental design for the short- and long-term assessment of targeted IL-1RA treatment. In experiment 3, long-term behavioural and histological outcomes of IL-1RA treatment were examined. In experiment 4, acute changes caused by PA and IL-1RA in microglia morphology were examined in the mPFC 24 h after PA. In experiment 5, gene expression changes caused by PA and IL-1RA were examined in mPFC via RNA sequencing analysis, 24 h post-PA. **B**, **C** 5-CSRTT reveals a rescue effect of IL-1RA on attention and learning deficits in adult PA animals (trial day *F*(2.203,96.95), *p* < 0.001; treatment *F*(3,44) = 3.421, *p* = 0.025; trial day × treatment interaction *F*(87,1276) = 2.786, *p* < 0.001). **D** The elevated plus maze (EPM) test shows no effect of IL-1RA on anxiety levels in PA animals (PA treatment *F*(1,50) = 4.242, *p* = 0.044, IL-1RA treatment *F*(1,50) = 0.022, *p* = 0.882). **E**–**H** Microglia morphology analysis 24 h post-PA in the PRL revealed a marked shaping effect of IL-1RA treatment on microglia soma size in PA animals (PA treatment *F*(1, 2050) = 2.810, *p* = 0.093; IL-1RA treatment *F*(1, 2098) = 2.875, *p* = 0.090; PA × IL-1RA interaction *F*(1, 2864) = 16.196, *p* < 0.001). Furthermore, PA led to decreased microglia branching (ramification index PA treatment *F*(1, 30.44) = 5.677, *p* = 0.017, IL-1RA treatment *F*(1,0.209) = 0.038, *p* = 0.843, PA × IL-1RA interaction *F*(1, 3.940) = 0.734, *p* = 0.391), unchanged by IL-1RA. In the IL, PA significantly increased soma size with no substantial shaping effect of IL-1RA (PA treatment *F*(1, 17783) = 15.043, *p* < 0.001, IL-1RA treatment *F*(1, 2875) = 2.986, *p* = 0.08, PA × IL-1RA interaction *F*(1, 173.14) = 0.179, *p* = 0.672). However, PA and IL-1RA treatment had a significant interactive effect on branch ramification (ramification index: PA treatment *F*(1, 25.8) = 4.555, *p* = 0.032, IL-1RA treatment *F*(1, 2.359) = 0.416, *p* = 0.518, PA × IL-1RA interaction *F*(1, 3887) = 4.556, *p* = 0.041). **I**, **J** Inhibitory synapse numbers showed significant increase in both PRL and IL of PA animals, while acute IL-1RA treatment showed a long-term therapeutic effect only in the IL (IL: PA treatment *F*(1,156) = 7.762, *p* = 0.005; IL-1RA treatment *F*(1,156) = 10.886, *p* = 0.001, PA × IL-1RA treatment interaction *F*(1,156) = 3.564, *p* = 0.06; PRL: PA treatment *F*(1,151) = 4.286, *p* = 0.04; IL-1RA treatment *F*(1,151) = 0.586, *p* = 0.44, PA × IL-1RA treatment interaction *F*(1,151) = 0.785, *p* = 0.376). Data regarding inhibitory synapses are shown as normalised bouton densities across experimental groups with median and quartiles on (**I**, **J**). 5-CSRTT 5-choice serial reaction time task, IL infralimbic cortex, PRL prelimbic cortex. Error bars represent mean + SEM; * PA treatment effect, # IL-1RA treatment effect, & PA × IL-1RA interaction effect.

To block IL-1 signalling, an endogenous competitive inhibitor, IL-1RA, was administered in a therapeutically relevant time window, at 1 h and 20 h after PA (Fig. 4A). To assess the long-term impact of IL-1RA on emotional and cognitive domains, we focused on evaluating anxiety and attention deficits in the EPM and 5-CSRTT tests, which represented the most substantially affected behavioural outcomes in our model. IL-1RA was able to prevent the attention deficit observed in adult PA males during the 5-CSRTT. Specifically, IL-1RA treatment increased the animals’ acquired stage above control level (Fig. 4B, C), proposing a protective effect of IL-1RA against PA-induced long-term attention and learning difficulties. IL-1RA administration did not affect anxiety levels as measured by time-percent spent in the open arm during the EPM test (Fig. 4D).

Acute microglia morphology analysis revealed significant effects of both PA and IL-1RA treatment in the PRL and IL regions of the mPFC (methodology established by Clarke and colleagues [61] and examining 62 parameters). In the PRL, IL-1RA had a reducing effect on microglia soma size in PA males (Fig. 4E). However, microglia branching was decreased in PA animals regardless of IL-1RA treatment (Fig. 4F). In the IL, PA led to a significant increase in microglia soma size (Fig. 4G). Additionally, IL-1RA treatment had a significant effect on branch ramification in PA animals (Fig. 4H).

Correlation analysis of morphological features with treatment groups confirmed that PA microglia exhibited larger somas. Conversely, properties describing ramification, along with the regression coefficients (slope of the linear regression between the logarithm of branch number and the logarithm of radius), were higher in the control group (Fig. S5A). Using a Random Forest classifier (RF), we identified soma size, maximum branch number and radii of maximum intersections as the most significant parameters for distinguishing treatment groups (Fig. S5B). Comparing the PA vehicle and PA IL-1RA groups, the average branch length, number of intersections, and regression coefficients showed the strongest correlation with the treatment (Fig. S5C). Furthermore, RF revealed lacunarity (a measure of inhomogeneity in the cell mask) as the most discriminating factor between groups, alongside various properties related to ramification and branching (Fig. S5D).

Histological analysis of VGAT puncta in adult males confirmed the increase in inhibitory synaptic terminals caused by PA. This was ameliorated by early IL-1RA treatment in the IL, but not in the PRL (Fig. 4I, J).

These results demonstrate that acute administration of IL-1RA can rapidly modulate microglial responses and influence long-term inhibitory balance and behaviour, proposing a specific and accessible therapeutic approach for PA-induced alterations.

### Transcriptomic profiling reveals molecular pathways acutely altered by PA

**Fig. 5:**
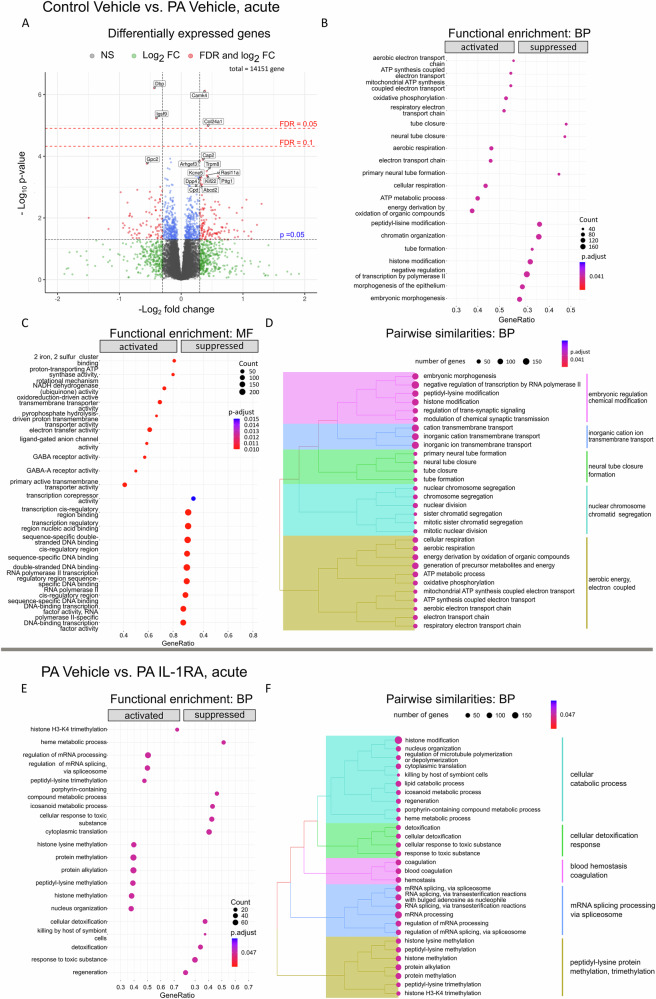
Acute molecular and pathway alterations following PA and IL-1RA treatment. **A** Experiment 5: Differential gene expression analysis of medial prefrontal cortex (mPFC) samples isolated 24 h after PA, demonstrating significant upregulation of *Dbp* and *Igsf9*, alongside downregulation of *Camk4* and *Col24a1*. **B** Functional gene set enrichment analysis utilising Gene Ontology (GO) Biological Processes reveals robust downregulation of pathways related to aerobic electron transport and metabolic processes, concurrent upregulation of pathways involved in neural tube formation, and altered epigenetic modifications. Note: ‘activated’ gene sets are enriched for genes with positive signed *p* values (upregulated); suppressed gene sets are enriched for genes with negative signed *p* values (downregulated) in the Control vehicle group compared to the PA vehicle. **C** Gene set annotations based on GO Molecular Functions, indicating significant alterations in ligand-gated anion channel and GABA receptor activities following the PA insult, confirming associated metabolic and transcriptional changes. **D** Hierarchical clustering analysis of pairwise similarities among significantly altered pathways, based on overlapping participant genes, highlighting dominant clusters related to aerobic energy transport, neurodevelopment, cell cycle regulation and membrane/synaptic transmission. **E** Effects of IL-1RA treatment on PA-injured animals, illustrating substantial alterations in post-transcriptional and post-translational modifications, as well as activation of detoxification pathways. Note: ‘activated’ gene sets are enriched for genes with positive signed *p* values (upregulated); suppressed gene sets are enriched for genes with negative signed *p* values (downregulated) in the PA vehicle group compared to PA IL-1RA. **F** Metabolic pathway changes resulting from IL-1RA treatment contribute to the observed rescue phenotypes in behavioural and histological analyses. Note: All labelled *p* values were corrected for multiple testing using False Discovery Rate (FDR, Benjamini-Hochberg method) corrections. Each panel includes relevant statistical analyses and annotations to emphasise the significance of the findings. Adjusted *p* values were calculated with the fgsea R package using the signed *p* values of all investigated genes in the experiment. Abbreviations: BP Gene Ontology: Biological Pathways, MF Gene Ontology: Molecular Functions.

To elucidate the acute molecular and pathway alterations following PA, we conducted RNA sequencing on mPFC samples isolated 24 h post-insult. Differential gene expression analysis revealed significant upregulation of the *Dbp* (D site of albumin promoter binding protein) and *Igsf9* (Immunoglobulin Superfamily Member 9) genes, the latter crucial for inhibitory synapse development. We observed downregulation in *Col24a1*, a collagen family member involved in extracellular matrix development. We also noted the downregulation of *Camk4*, which encodes calcium/calmodulin-dependent protein kinase type IV, a key mediator in calcium signalling essential for neuronal and immune cell function (Fig. 5A).

Functional gene set enrichment analysis revealed robust suppression of pathways related to aerobic electron transport and metabolic processes by PA, alongside activation of pathways involved in altered epigenetic modifications (Fig. 5B). Significant alterations were observed in ligand-gated anion channels and GABA receptor activities (Fig. 5C). Hierarchical clustering analysis of pairwise similarities among significantly altered pathways identified dominant clusters related to aerobic energy transport, neurodevelopment, cell cycle regulation and membrane/synaptic transmission (Fig. 5D). Finally, IL-1RA treatment in PA animals resulted in substantial alterations in post-transcriptional and post-translational modifications, and activation of detoxification pathways (Fig. 5E). These changes were accompanied by metabolic pathway alterations (Fig. 5F), which may contribute to the rescued phenotypes observed during behavioural and histological analyses.

## Discussion

This study explores the contribution of acute neuroinflammatory mechanisms to the long-term neuropsychiatric consequences of PA with implications for early targeted therapeutic interventions. We utilised a rodent PA model based on the foundational work of Ala-Kurikka et al. [45, 46], partially modifying it to align with our objectives to explore long-term behavioural consequences. This model is translationally relevant to near-term human patients, taking into account neocortical development [62–65] and the pathophysiological changes in acid-base balance during PA [46–48]. Our PA model does not involve carotid ligation and thus does not induce focal injury or surgery-related inflammation, which would shift the neuroimmunological characteristics of PA towards directions less conducive to translational interpretation [66, 67].

We used a top-down approach to study the consequences of PA. Extensive behavioural assessment in both sexes was followed by histological analyses, comprehensive gene expression profiling and pharmacological intervention in male animals. Behavioural data from males revealed specific neuropsychiatric deficits, highlighting cognitive impairments, decreased attention and learning performance, alongside emotional disturbances, increased anxiety and impulsivity. The assembly of these dysfunctions may be interpreted as a shift towards an ADHD-like phenotype [68], a neurodevelopmental disorder frequently comorbid with anxiety disorders in human patients [69]. This phenotype has repeatedly been suggested as a potential consequence of PA [4, 70–74]. Hence, our model may better capture the heterogeneity of clinical outcomes observed in PA survivors, reflecting a range of impairments- from subtle behavioural deficits to more pronounced dysfunction- rather than the uniformly severe alterations typically induced in standard preclinical models [75–78].

The perinatal period is a critical phase of increased CNS vulnerability, fundamental for healthy synaptogenesis, GABAergic signalling, gliogenesis and myelination [79]. Previous studies on PA have primarily focused on higher-order processing regions, such as the frontal cortical areas, hippocampus and amygdala [80], all heavily implicated in neurodevelopmental disorders [81, 82]. The mPFC is a key region for top-down control of various higher-order cognitive processes, such as decision making, emotional regulation and attention [39, 40]. Our model resulted in a lasting increase in mPFC inhibitory synapses, while preserving neuronal and excitatory bouton numbers in male animals. Disturbances in inhibitory boutons of the mPFC have been associated with deficits in attention, impulse control and anxiety-like behaviours [83, 84]. Such alterations could lead to broad neuropathologies in a critical period of plasticity, potentially contributing to the neuropsychiatric consequences of PA. Our observations align with literature suggesting that inhibitory interneurons are particularly vulnerable to perinatal injuries and hypoxia due to their high metabolic demands and reliance on oxidative phosphorylation [85–89]. This supports the characterisation of neurodevelopmental disorders as ‘interneuronopathies’ [68, 90], emphasising GABAergic developmental and circuit alterations as key histopathological features [91, 92]. Furthermore, microglial responsiveness during development is crucial for the refinement of neuronal circuits, particularly those involving GABAergic signalling [93], which becomes critical under pathological conditions.

Inflammation plays a key role in the perinatal programming of neurodevelopmental disorders [94]. Microglial morphology, closely linked to transcriptomic and proteomic changes [35], serves as a sensitive marker of inflammation. We examined acute PA-induced morphological changes in microglia of males, and the observed inflammatory transformation supports our model’s applicability for investigating mechanisms of moderate PA. Disturbed inflammatory niches could interfere with neurodevelopmental programmes. Microglia influenced by early-life stress have been demonstrated to lose their long-term synaptosome phagocytotic function, leading to an increase in synaptic terminals [95]. Furthermore, GABA-receptive microglia have been shown to selectively interact with inhibitory synapses exclusively in the postnatal period [96]. It could be hypothesised that PA leads to a loss of this complex and specific function, affecting inhibitory synapse development more prominently.

It remains unclear whether microglial IL-1RA, which has been implicated in the molecular mechanisms of the inflammatory response [97], plays a role in PA-related inflammatory changes or if IL-1RA targets other cell types in this experimental model. Nonetheless, IL-1-induced neuroinflammation is a well-established contributor to oxidative stress and neuronal damage [27]. Our findings support this notion, demonstrating that pharmacological blockade of IL-1R resulted in altered microglial morphology in the mPFC and partial amelioration of histological and behavioural sequelae, particularly learning and attention in males. It is possible that IL-1RA influences cognition, without significantly impacting emotional behaviours like anxiety in this model. This domain-specificity may be attributed to regional differences in microglial functional characteristics, actions of IL-1 on other cells or the differential influence of IL-1-signalling on circuitry-specific processes, in line with brain regionspecific differences in therapeutic sensitivity to IL-1RA. Also, distinct circuitries are involved in regulating these behavioural domains. Attention and executive control are heavily reliant on mPFC-thalamic-striatal circuits, where IL-1 receptor expression follows a highly organised pattern in adult rodents [98], and interleukins have been suggested to modulate working memory and executive functions through effects on synaptic plasticity, neuroinflammation and neuronal excitability [99]. In contrast, anxiety-like behaviours are more strongly governed by amygdalar-hippocampal-hypothalamic networks [100], where IL-1 signalling may play a different role, potentially involving neuromodulatory systems. The developmental stage of these circuits in our model adds further complexity, as neuroimmune interactions may evolve with maturation. In addition, the density and activation profile of microglia, as well as the sensitivity of local circuits to IL-1RA, may vary substantially across these regions [101]. These differences may underlie the behavioural selectivity we observe. Further research is needed to clarify how IL-1 modulates functionally distinct circuits and to identify brain region-specific therapeutic sensitivities to IL-1RA.

Since inhibition of IL-1R is already employed in the management of inflammatory disorders [102, 103], inhibiting microglial IL-1 signalling appears to be feasible and translationally relevant for the mitigation of neuroinflammation in the context of PA. Previous preclinical studies on different models of early life disease have demonstrated that IL-1RA can effectively reduce inflammation and tissue injury, without reported side effects [104, 105]. However, the immune-modulating properties of IL-1RA warrant careful consideration, particularly in the context of neonatal immune vulnerability. In adult human patient populations, although IL-1RA is generally well tolerated, skin irritation and infectious complications have been reported, particularly with long-term use or in individuals with compromised immunity [106]. Importantly, a recent clinical trial has begun to assess the safety, feasibility and pharmacokinetics of IL-1RA administration in very preterm neonates, which will help guide future applications in the perinatal context [107].

Additionally, data from female subjects highlight the frequently observed sex-dependent susceptibility to PA-induced neuropsychiatric deficits [108]. This variability may be attributed, in part, to sexual dimorphism in inflammatory responses and microglial function in the perinatal period [109, 110].

Analysis of differentially expressed genes in the mPFC of PA males underpinned marked acute synaptic deregulation. Notably, upregulation of *Igsf9* is known to be induced by hypoxia in neural progenitor cells. Conversely, PA resulted in downregulation of *Col24a1*, an extracellular matrix (ECM) component, potentially disrupted by PA. Both are established regulators of axonal outgrowth, synaptic differentiation and inhibitory synapse maintenance [111–113]. Also, *Camk4* downregulation has been linked to disrupted synaptic plasticity, learning deficits and anxiety in rodents [114, 115]. Furthermore, the IL-1β has been shown to cause deregulation of the circadian rhythm-related transcriptional activator gene *Dbp* [116, 117]. Upregulation of *Acss2* may play a crucial role in Hypoxia-Inducible Factor 2 acetylation under hypoxic conditions [118]. Our comprehensive gene set enrichment analysis indicated PA-induced adjustment in mitochondrial energy transport and ATP metabolism and alterations in cell cycle regulation, aligning with previous literature [119, 120]. Notably, the analysis highlighted changes in GABA-related signalling, supporting our findings on increased sensitivity of inhibitory synapses to PA.

Recent literature shows that the extent and nature of long-term transcriptomic changes caused by PA appear to depend on injury severity and compensatory mechanisms over time [121]. Our pathway analysis in adult males revealed subtle alterations in epigenetic regulation, along with changes in immune and signalling pathways. These findings support the idea that long-term effects of PA are mediated largely by epigenetic mechanisms, which can influence gene regulation without necessarily producing large, sustained changes in steady-state mRNA levels [122]. Epigenetic modifications have been implicated in activity-dependent gene regulation during critical periods of brain development, and their dysregulation may contribute to persistent changes in neuronal function and behavioural outcomes [123]. Furthermore, early-life stress may increase vulnerability to subsequent challenges in adulthood [124], implying that differences in gene expression may potentially emerge following a secondary stressor, highlighting the importance of investigating such effects in future studies.

Acute IL-1RA treatment led to notable alterations in post-transcriptional and post-translational processes, including histone modification, mRNA splicing and protein methylation. PA may trigger key regulators of cellular response to hypoxia, such as Hypoxia-inducible factor 1 alpha (HIF-1α), which interacts with histone acetyltransferases and deacetylases to modulate chromatin accessibility [122]. Additionally, NF-κB-mediated inflammatory responses, which are closely linked to chromatin remodelling, are induced by IL-1β, proven to increase after PA [36, 125]. IL-1β also regulates RNA-binding proteins and splicing factors, disrupting normal mRNA processing under hypoxic stress [126]. These pathways may be partially suppressed by pharmacological IL-1 inhibition. The fact that IL-1RA treatment modulated these processes supports the hypothesis that neuroinflammatory signalling can influence epigenetic modifications and suggests that IL-1RA engages distinct regulatory mechanisms, rather than directly counteracting the pathways disrupted by PA.

Pathological hyperperfusion changes, derived from neuroimaging of blood flow changes 24 h after the PA insult, are known to trigger radical and nitric oxide-driven damage and inflammation [127]. Interestingly, excessive relative perfusion was observed in brain regions critical for cognitive and emotional functioning, characterised by high metabolic activity during development, rendering them particularly vulnerable to hypoxia [128, 129]. In line with this, normal microglial function is essential for adaptation to cerebral hypoperfusion [38], which may be disturbed by inflammatory transformation. Hence, acute perfusion neuroimaging may be a valuable prognostic tool in cases of mild to moderate PA [130].

PA resulted in an acute burst in stress-related hormones, demonstrating its capacity as an early-life stress paradigm that activates the HPA axis [47]. Intriguingly, at P7 we observed reduced levels of corticosterone alongside aldosterone and ACTH increase, underscoring the distinctive characteristics of the neonatal stress-hyporesponsive period, during which aldosterone is implied to play a key role in the hormonal stress response [131].

This study has its limitations in the representation of peripartum adaptive mechanisms and immunological milieu that certainly affect the course of PA in human neonates. Moreover, future studies combining male and female animals in a single cohort should formally test interactions between sexes to provide formal evidence of the differences in behavioural alterations. In addition, females were not evaluated for oestrous cycle phases during behavioural testing; doing so would have been challenging without disrupting the integrity of complex, multi-day behavioural studies. Incorporating such assessments will be essential for obtaining a thorough understanding of how oestrous cycle phases influence behaviour in future research. Our focus was primarily on the mPFC, driven by the observed behavioural consequences of PA. Other critical brain areas and circuits involved in higher-order processing, and cell type-specific analysis of gene expression changes warrant further investigation.

In conclusion, we employed a translationally relevant rodent model of mild to moderate PA that provides a means to investigate the subtle neuroinflammatory mechanisms underlying behavioural alterations that manifest later—specifically in male animals—and closely resemble those observed in human patients. Our findings highlight both the short- and long-term therapeutic effects of acutely administered targeted anti-inflammatory treatment (IL-1RA) on the histological and behavioural consequences of PA in males. These results suggest that IL-1RA may serve as a promising therapeutic option for the early prevention of immune-mediated cognitive deficits, useful for optimising treatment strategies for PA in clinical settings.

## Supplementary information

Supplementary material

## Data Availability

The datasets generated and analysed during this study are available from the corresponding author upon reasonable request. RNA sequencing data have been deposited in NCBI’s Gene Expression Omnibus and are accessible through GEO Series accession number GSE295589.
