# Supplementary material for: Inflammatory mechanisms contribute to long-term cognitive deficits induced by perinatal asphyxia via interleukin-1
Source: Neuropsychopharmacology. 2025 Oct 2;51(2):440–54. doi: 10.1038/s41386-025-02227-8 (PMC12708856; doi:10.1038/s41386-025-02227-8)
Supplement: Supplementary file 1 — Supplementary material [file 41386_2025_2227_MOESM1_ESM.docx]

# SUPPLEMENTARY MATERIALS

## Materials and Methods

**Animals**

Rats were housed in temperature (24±1 °C) and humidity (50±10 %) controlled rooms under a 12-hour light-dark cycle, with ad libitum chow (Special Diets Services, VR1) and water. Breeding pairs were cohoused for 5 days, and dams were isolated for the remainder of pregnancy. Litters were culled to 8-10 pups per litter on postnatal day 0 (P0) to minimise maternal care variations. Pups were individually marked on P3 with a green tattoo (Micro-Tattooing System, Harvard Apparatus, USA; Ketchum Animal Tattoo Ink Paste, Canada) for identification. After weaning (P21), animals were group-housed (3-4 rats per cage, Tecniplast 1291H Eurostandard III H) with same-age, same-sex and same-treatment mates, in a reverse circadian light-dark cycle room (lights on at 7 PM, off at 7 AM) and habituated to experimenters for one week before behavioural testing.

### Perinatal Asphyxia Insult (PA)

At P7, male and female pups were separated dams, weighted, and randomly assigned to treatment chambers (30x15x20 cm) with perforated plexiglass dividers for individual isolation during PA and recovery. Chambers were placed in an isothermal water bath (35.6 °C; WNB 45, Memmert, Germany). Based on pilot experiments, the pups were kept for 45 minutes to equilibrate their core temperature to normothermia (37.0-37.5 °C).

Thereafter, treatment chambers were filled with a PA-inducing gas mixture (4% O_2_ and 20% CO_2_ in N_2_; Messer Hungarogáz Ltd, Hungary), with continuous O_2_ and CO_2_ concentration monitoring (TR250Z Oxygen Sensor, K-33 BLG Sensor, CO_2_Meter Inc., USA). After 15 minutes of exposure to the PA-inducing gas mixture, the chambers were opened and ventilated to restore normoxia. The pups were allowed to recover from the insult for 45 minutes before returning to dams. PA resulted in 25-30% mortality, with no sex differences. No further mortality occurred among pups. Control pups were separated from dams for the same duration in chambers filled with room air throughout the experiment (Fig. 1A). Animals were semi-randomly assigned to experimental groups within litters, using alternation to avoid litter effects and ensure balanced group sizes.

### Behavioral testing

Juvenile and adult behavioral experiments were performed during the first half of the active (dark) cycle in a separate experimental room. To minimise confounding effects and carry-over between tests, the assessment started with the less stressful tests and ended with those recquiring food-restriction (e.g., 5-CSRTT). To adhere to ethical guidelines and reduce animal use, each animal participated in multiple tests, ensuring comprehensive assessment without overtesting. The early neurodevelopmental tests, playfight, accelerating rotarod, social interaction test, resident-intruder test, and Y-maze tests were analysed manually in a blind manner or by Solomon Coder (<https://solomon-coder.software.informer.com/download/>.), while the hole-board, open-field, elevated plus maze, sociability and social recognition, contextual fear learning and Morris water maze tests were analysed with EthoVision XT 13 or 15 software (Noldus Information Technologies, Wageningen, The Netherlands). The delay discounting, 5-CSRTT, set-shifted 5-CSRTT and Go/no-Go tasks were performed in automated operant chambers (Med Associates, USA). The testing tools were cleaned with soapy water or 70% ethanol between the trials and dried with a paper towel.

In cognitive tests performed in automated operant chambers, the feeding of the subjects was restricted throughout the testing period to increase their motivation for food rewards (45 mg, Dustless Precision Pellet F0021, Bio-Serv, USA). Four days before the operant tests the animals were separated and kept individually for precise dosage of the amount of the restricted food pellets. Animals were weighed every three days and their food was adjusted to keep them at 85-95% of their original body weight. Females were not assessed for estrous cycle phases during behavioral testing as this would have been difficult to achieve without disturbing complex behavioural studies. However, behavioural assessments extended over multiple days likely encompassing all stages across the testing period. At the end of behavioural assessments, termination with CO_2_ was performed on those animals who did not take part in histological studies.

#### Early neurodevelopmental testing for robust neurological defects

Pups underwent neurological tests for gross motor assessment and ultrasonic vocalization measurement at age P8 (24h after the PA insult). The number of animals tested was 15-16/ treatment group.

**Surface righting reflex test**: To investigate the intactness of the locomotor and vestibular systems^1^, the pups were placed in supine position on a supporting surface. The time needed to return to their normal prone position with all paws on the surface (max. 30 sec) and the side of the turning was registered.

**Negative geotaxis test**: To assess the sensorimotor integrity, coordination and vestibular response of the pups^2^, they were placed on an inclined board at 45^o^ (30x30 cm) covered by cloth paper facing downward. The time needed for the pups to rotate 180o and take an uphill position (max. 45 sec) was recorded and analysed.

**Measurement of ultrasonic vocalization (USV)**: To measure separation-induced US vocalization, a common test of neonatal emotional function^3^, pups (P8) were individually placed in a glass beaker (Ø 12 cm, 18 cm high) without bedding or heating for 10 minutes. All animals were tested under identical environmental conditions, and the influence of ambient temperature on vocalisation was consistent across groups. Ultrasonic microphones (CIEL-Electronique CDB 205 R2) placed 10 cm above the beaker converted the vocalizations to audible sounds. Audacity 2.0.5 (Creative Commons, USA) free software was used to register the time-pattern (frequency and cumulative duration) and spectrum of the vocalizations. The recordings were later analysed by a Rat Call Counter, developed by S. Zsebők^4^. The threshold value was set at a signal amplitude of 0.4 mV to exclude background noise.

#### Juvenile emotional and cognitive testing

**Playfight test:** Playfight test was performed at the age of 26-28 days to examine the animals’ early social and aggressive behavioral patterns^5^. The number of animals tested was 10/ treatment group. After two days of 10min individual habituation to the test room and cages, the animals were isolated for 3.5h in standard home cages and then placed in the testing cage in pairs, and video recorded for 10 minutes. Rats of the same sex and treatment but unfamiliar to each other were paired. One of the animals was marked with semi-permanent dye for easier differentiation one day prior to testing.The frequency and duration of exploring, sniffing, grooming, pouncing, pinning, jabbing, kicking or defence were measured using an event recorder software based on the criteria previously described by Veenema and colleagues^6^.

**Hole-board test:** At adolescent age (P30-35) the rats took part in a hole-board test to examine their exploratory behavior and spatial learning abilities^7^. The number of animals tested was 15-16/ treatment group. Before the test, the animals were food-deprived for 1h to increase their motivation. On the habituation day the animals were placed in the test boxes for 10 min to explore the arena (79 × 54 × 35 cm, black plastic box) that had 16 uniformly positioned round holes in its floor. On days 2-4 (learning phase) every hole was filled with one sugar pellet (45 mg Dustless Precision Pellet F0021, Bio-Serv, USA) and the edge of each hole was marked with a piece of white adhesive tape. This phase took 5 min or less if the animal ate all the pellets in a shorter time. On days 5-6 (test phase, 5 min or less) only four holes were filled and marked and the animals were expected to learn that only the marked holes contain food pellets. On days 7-8 (reverse learning test phase) four other holes were filled and marked to examine the animals’ ability to flexibly associate the newly marked holes with the reward. The latency to eat all pellets and the number of correct and incorrect nose pokes were measured (the latter two only in reverse learning phase).

**Y-maze test:** The Y-maze is a behavioral test that measures the willingness of rodents to explore new environments and is widely used for testing spatial working memory^8^. The number of animals tested was 15-16/ treatment group. The subjects (P24) were placed in a Y-shaped grey plexiglass maze (arm 45 × 17 × 30 cm, the angle enclosed by the arms is uniformly 120°) for 5 minutes, with the test-room brightness adjusted to ≤ 60 lux. The percentage of spontaneous alterations was later analysed. A spontaneous alternation was defined by entering into the three arms consecutively without repeated same-arm visits. The alternation index was defined as the ratio of actual and possible alterations (defined as the total number of arm entries minus two) multiplied by 100 as shown in the following equation: Alternation% = [Number of alterations/(Total arm entries − 2)] × 100.

#### Adult behavioral tests

**Rotarod test:** The rotarod test is used to assess the sensorimotor coordination and motor learning in rodents^9^. Here, on day 1 (training day) the rotarod (IITC, USA) was set to a constant rotation speed of 4 rpm and the animals were placed on the rod for a maximum of 5 times until they were able to stay on it for 180 sec; Thirty-minute intertrial intervals were applied between attempts. On day 2 (test day) the rotarod was set to an accelerating speed of 4 to 25 rpm in 180 sec and the animals’ latency to fall was measured. The brightness in the room was adjusted to 70 lux or less during the experiment. The number of animals tested was 9-12/ treatment group.

**Open field test:** Open field (OF) test was performed at a young (P33, N= 5-9/ treatment group) and older adult (P87, N= 6-11/ treatment group) age under infrared lighting to evaluate the locomotion, exploratory behavior and anxiety state of the animals^10^. The distance travelled, as well as the number of entries and time spent in the center zone (50% area of the whole arena) of the OF box were analysed during the 10-minute trial period. At age P33 the size of the black coloured OF apparatus was 79x54x35 cm, at age P87 a circular OF arena was used (100 cm Ø, 35 cm high wall).

**Elevated plus-maze test:** The elevated plus-maze test (EPM) was performed to assess the locomotion and anxiety state of the animals by analysing the total distance travelled, the number of entries and time spent in the open arms during the 5-minute trial period^11^. The number of animals tested was 6-11/ treatment group. The subjects were placed in the central area of a plus-shaped maze facing a closed arm of the grey plexiglass EPM apparatus, which was elevated to a height of 70 cm, each arm having 42x12 cm floor space, and the closed arms having 35 cm high walls; infrared lighting was used during testing.

**Delay discounting procedure:** The delay discounting procedure (DD) measures the impulsive behavior of rodents^51^. The number of animals tested was 6-11/ treatment group. The procedure was performed on food restricted animals similarly to that employed by Adriani et al.^12^ and adopted to our automated operant chamber system (Med Associates, USA). The DD procedure started with a training phase (30 min daily for 5 days) in which subjects were trained to respond on one of the two nose poke response holes placed on the operant chamber wall in order to receive a ‘small reward’ of one sugar pellet (45 mg Dustless Precision Pellet F0021, Bio-Serv, USA), while response on the other nose poke response hole resulted in the delivery of a ‘large reward’ of 5 sugar pellets. Rewards were delivered to a pellet delivery receptacle placed between the two nose poke holes. The side of the ‘large-reward’ hole was randomly assigned in order to avoid side-preference. Each subject was placed in the same chamber with a constant hole assignment throughout the entire testing period. Chamber light was switched on for a 25 s time-out (TO) period following a response on any of the holes, making the chamber light a cue associated with a correct response. During the TO period nose-pokes were recorded but not rewarded. At the end of the training phase, subjects showed a preference for the ‘large reward’ nose poke response hole in approximately 90% of all trials. In the test phase (30 min daily for 8 days) a delay was inserted before ‘large reward’ delivery. The length of the delay was progressively increased daily (10, 20, 30, 45, 60, 80, 100 and 120 s). ‘Small rewards’ were delivered immediately after nose poke. In this phase, subjects were expected to gradually switch their preference from the delayed ‘large reward’ to the immediate ‘small reward’. During the training phase, the learning capacity of the animals was assessed by analysing the increase in large reward preference. In the test phase, impulsive behavior was indicated by the increased preference of the small immediate reward. The number of inadequate responses was also recorded, representing premature reactions during time-out and delay periods reflecting motor impulsivity, the inability to inhibit inappropriate actions.

**Morris water maze test:** To study the spatial learning and memory of the rats, a modified Morris water maze test (MWM) was performed^13^. A black coloured, round shaped tank (Ø180 cm, 60 cm high) was filled with tempered tap water (23±1°C), with a water level 1.5 cm higher than the hidden escape platform (Ø12 cm, 43 cm high). The apparatus was divided into 4 virtual quadrants, and the platform was placed in the middle of one of the quadrants. The walls of the experimental room were decorated with visual cues and the test room was illuminated by dimmed light (approx. 60 lux)^11^. The number of animals tested was 10-12/ treatment group. Animals were placed in the pool at one of the five release positions for four daily trials with 15-second inter-trial intervals (ITI) for five consecutive days. The order of releasing positions was randomised daily but kept constant between subjects. Rats were placed into the water facing the tank wall and were allowed to swim freely. Each trial continued until the animal located the hidden platform, with a maximum duration of 90 seconds. If the animal failed to find the platform within this time, it was gently guided to it. After reaching the platform, all subjects remained there for 15 seconds before being moved to the next starting point for the following trial. Rats were thoroughly dried with clean cotton towels after the swimming sessions. The average daily time taken to find the platform (escape latency) was used as a measure of learning performance.

**Five choice serial reaction time task procedure:** The five choice serial reaction time task procedure (5CSRTT) is widely used to measure attention, impulsivity and cognitive abilities in rodents. In our experiment, a 12-stage protocol was performed, as previously described by Bari and colleagues^14^. The number of animals tested was 6-7/ treatment group. Briefly, adult rats were tested during 30 minutes daily session for six weeks, each week consisting of five consecutive test days followed by a two day resting period (approx. 30 sessions/subject in total). During sessions, animals were trained to detect brief visual stimuli (LED light) that appeared in a random order inside one of the five nose poke response holes placed on the chamber wall and make a nose poke response at the illuminated hole. A nose poke in such ’rewarded’ response hole during the light cue or the following pre-determined time interval (limited hold) resulted in the delivery of a sucrose pellet into a receptacle located on the opposite wall of the chamber. Response errors were represented by the number of omissions (when no response was made during the trial), incorrect responses (responses at a non-illuminated hole) and premature responses (response before the visual cue switched on), all of which were punished by a 5 second dark period. If subjects fulfilled the predetermined criteria for the given stage (for description of criteria see Bari et al.), they were subjected to the next upper test stage at the following daily session. Cue and limited hold length progressively decreased with sessions while criteria became progressively more difficult to fulfill. The average of the highest stage reached, response accuracy and the number of sessions needed to reach a particular stage were analysed across treatment groups. During the testing period subjects were kept on a restricted diet in order to increase their motivation to perform in the task for sucrose pellet rewards. Food restriction was initiated 3 days prior to the first test session and food amount was adjusted to maintain 90% (±5%) of initial body weight.

**Set-shifted 5-CSRTT procedure:** Adult rats (N=11-15/ treatment group) were tested in 2 test phases to measure operant learning, attention and cognitive flexibility. Animals were tested for 30 minutes daily for six weeks in automated operant chambers. In Phase 1, subjects were trained to respond in either the 2nd or 4th nose poke response hole out of 5 holes on the wall of the operant chamber set up in a similar manner as described at the 5CSRTT. At the beginning of every trial a cue light randomly illuminated one of the five nose poke response holes which was an irrelevant stimulus and had to be ignored by the subjects. Correct response was rewarded with a sucrose pellet. The position of the rewarded hole was constant during this phase and was randomly assigned across subjects in order to prevent side preference. If the subjects acquired ≥80% accuracy (ratio of correct out of all responses) on two consecutive days, the test was continued in Phase 2. This phase was identical to the first stage of 5CSRTT described above.

Go/no-Go task: To measure response inhibition, a Go/no-Go task^15^ was performed for three weeks. Animals (N=7-9/ treatment group) were trained in two sequential task-phases in automated operant chambers (Med Associates, USA) for 30 min each daily session. In the first (’Go’) phase, subjects were trained to make a nose poke response to a light cue (’Go’ stimulus) at a predetermined rewarded hole of two nose poke response holes on the wall of the chamber in order to receive a reward pellet. If the subjects reached >80% response accuracy on two consecutive days, the second (’Go-no-Go’) phase began. In this phase, besides the ’Go trials’ described above, ’no-Go trials’ were presented, during which an auditory -cue (’no-Go’ stimulus) was added to the visual ’Go’ cue. During ’no-Go’ trials omission of response was rewarded while responding did not result in reward delivery. ’Go’ and ’no-Go’ trials were presented in a random order with a maximum of three consecutive trials of the same type. Response accuracy was separately recorded for ’Go’ and ’no-Go’ trials.

**Social interaction test:** For the social interaction test^16^, animals of identical treatment group (N=7-9/ group), same sex and similar body weight (less than 10% body weight difference) were assigned in pairs. The stimulus rats were completely unfamiliar to the experimental animals, and originated from a different litter. This ensured that the interaction reflected social exploratory behaviour rather than recognition of familiar conspecifics. One of the subjects was marked with semi-permanent dye for easier differentiation two days prior to testing. The animals were separately habituated to novel test-cages filled with clean bedding for 10 min on two consecutive days before the test. On the test day the pairs were placed together in the unfamiliar testing cages for 10 minutes under low light conditions, behavior was video recorded and their resting, exploring, grooming, sniffing, offensive/ defensive and biting behavior were scored later by an experimenter blinded to treatment groups.

**Sociability and social recognition test:** To further study the social behavior of the rats^17^, they took part in the sociability test performed in a 3-chamber-sociability box (central chamber 30x50x40cm, lateral chambers 50x50x40cm), which contained two empty cylinders with perforated, transparent walls on the left and right sides. 11-12 animals were tested from each treatment group. First, the animals were habituated to the test-box for 10 min, during which they explored the chambers and were expected to be equally interested in both cylinders. After completing the habituation, the animals were placed into clean bedded cages for 3 minutes and an unfamiliar juvenile rat (P28-P32) of the same sex was placed inside one of the cylinders. During the second (sociability) phase the animals were allowed to freely explore the apparatus for 10 minutes. The behavior of the animals was video-recorded and analysed afterwards. Sociability index was calculated as (time spent in the ‘social’ chamber)/(time spent in the ‘social’ chamber + time spent in the ‘empty’ chamber).

**Resident- intruder test:** To measure territorial aggressive behavior, resident-intruder test was performed^18^. 3 days prior to testing the animals (N= 11-12/ treatment group) were transferred to individual cages with clean bedding to evoke their territorial behavior. On the testing day a smaller sized, unfamiliar male opponent (intruder) was placed into the cage of the tested (resident) animals for 20 minutes. The tests were video-recorded and then analysed blindly by an experienced researcher, taking into consideration the latency, frequency, location, and intensity of bites.

**Contextual fear learning:** For contextual fear conditioning, female rats (N=11-15/ treatment group) were placed into a clear Plexiglass chamber (40 × 40 × 40 cm) with an electrical grid floor (Coulbourn Instruments). Fear conditioning was performed at maximum light intensity for 7 minutes, delivering 10 shocks of 2.4 mA after a 2.5 min habituation period. Long-term contextual fear recall (Context A) was tested for 5 minutes in the same experimental room, equipment and by the same experimenter 28 days after fear conditioning. Fear extinction (Context B1 and B2) was assessed on the following days for 20 minutes in a completely different setting (different room, illumination, equipment, experimenter)^19^. Time spent freezing was analysed. Software parameters and thresholds were optimised to reach R> 0.9 correlation with treatment-blind manual scoring.

For downstream analyses such as immunohistochemistry and transcriptomic profiling, we focused on male animals, as behavioural alterations were predominantly observed in this group following perinatal asphyxia. Given our aim to characterise mechanistic changes associated with the observed phenotype, we prioritised analysis in the group where a robust behavioural effect was detected.

### Immunohistochemical Studies

#### Fixation and Tissue Processing

For histological studies, a subset of animals was deeply anaesthetised with isoflurane (P8) or a mixture of ketamine (75 mg/kg) and xylazine (15mg/kg; adults). The animals were perfused transcardially with 0.1 M phosphate-buffered saline (PBS) and 4% paraformaldehyde (PFA) in PBS (pH 7.4). Brains were removed and post-fixed overnight in 4% PFA in PBS. For tissue processing, brains were cryoprotected in 30% sucrose-PBS for 48 hours at 4 °C. 30 μm sections were cut using a freezing sliding microtome and stored in cryoprotectant solution (50% sodium phosphate buffer, 30% ethylene glycol, 20% glycerol) at -20 °C.

#### Fluorescent Immunostaining and Image Analysis

Free-floating brain sections (N= 5 / treatment groups) were washed in Tris-buffered saline with Tween-20 (TBS-T) and underwent antigen retrieval in 10 mM boiling sodium citrate buffer (15 minutes, 85°C). After washing, sections were blocked in 5% normal goat or donkey serum in PBS for 1 hour at room temperature (RT), then incubated overnight with primary antibodies (*Supplementary Table 1*) diluted in 5% normal goat or donkey serum. On day 2, after washing and blocking with 5% normal serum, sections were incubated for 1 hour with a mixture of fluorescently conjugated secondary antibodies (*Supplementary Table 2*) at RT. Following washing, sections were mounted using Mowiol 4-88 (Merck, Germany). Images were acquired with a Nikon C2 confocal microscope (Plan Apo VC 20x DIC N2, NA = 0.75, xy 0.18 μm/pixel). Image analysis was performed using Ilastik 1.4.0 software for pixel classification^20^ and Fiji software^21^ for particle analysis. Custom macros were developed for the quantification and colocalization analysis of synaptic puncta acquired at 60x magnification. Custom macros automated background subtraction, Z-stack splitting and puncta detection or colocalization around cellular somas using intensity- and size-based filtering.

#### Analysis of Microglial Morphology

15-step Z-stack images were acquired using a Nikon C2 confocal microscope with a Plan Apo VC 20x DIC N2 objective (NA = 0.75) and a pixel resolution of 0.3 μm in the xy-direction, with 0.5 μm z-step size. Images were obtained from the infralimbic (IL) and prelimbic (PRL) regions of control and PA groups (N=6-7/ treatment groups). For analysis, an open-source microglial morphology analysis pipeline was used^22^. An object mask size of 300 μm² was applied. Pearson's r correlation coefficients were calculated to assess the target correlations between variables and group identity. RandomForest classification modelled the relationship between variables and group membership (control vehicle vs. PA vehicle and PA vehicle vs. PA IL1-RA). Before analysis, all 40 variables describing morphological aspects of microglial cells were standardised and feature importance was derived from RandomForest models with 100 estimators through a bootstrapping procedure consisting of 30 iterations of independent subsampling and model training. Analyses and visualisations were performed using Python (https://www.python.org/) and Sklearn (<https://scikit-learn.org>). All features are presented on panels A and C of Supplementary figure 5.

### Acute hormone measurements

For the estimation of acute hormonal changes caused by PA, trunk blood was collected from pups at P7 in baseline conditions (less than 5 minutes after separation from dam) and after PA (0h, 1h, 4h and 24h post-PA, N=6-7/ timepoint). The animals were rapidly decapitated, blood was sampled in ice-cold EDTA-containing tubes, centrifuged (2000 g, 20 min, 4 ^o^C), and plasma was stored at -20 ^o^C. ACTH, corticosterone and aldosterone concentrations were determined by radioimmunoassay (RIA), measuring all samples in the same assay. ACTH and aldosterone assays followed the protocol of Zelena et al.^23^ Corticosterone, was pleasured using a method of Toth et al.^24^ A specific rabbit antiserum against corticosterone-3-carboxymethyloxime (prepared in our Institute in rabbits), and 125I-labeled carboxymethyloxime–tyrosine–methyl ester derivative tracer (catalogue no. I-RBO-36, Institute for Isotopes, Budapest, Hungary) were used.

### SPECT and MRI Imaging

The effect of PA on cerebral blood volume was assessed 24 hours post-PA (N= 4-6 animals/ treatment group/ imaging method) by dextran-coated iron oxide nanoparticles determined by 1 Tesla MRI volumetry^25^ (coronal/vertical T1-weighted gradient echo sequencing, 80 axial slices of 0.3 mm thickness, 200 × 200 pixel, TR/TE/FA 11.2/2.1/65; nanoScan 1T PET/MRI; Mediso Ltd., Budapest, Hungary). Manual delineation of brain regions was performed using vivoQuant 1.22 software (inviCRO-Konica-Minolta Inc., Boston, US). Brain perfusion was measured by Single-Photon Emission CT (SPECT) imaging with 99mTc-HMPAO (Hexamethylpropyleneamine Oxime; Medi-Radiopharma Ltd., Budapest, Hungary). HMPAO was labelled with eluated 99mTc (Ultra-Technekow Technetium Generator, Mallinckrodt Medical, Petten, Netherlands) and physiological saline was injected in 74.5+/-8.9 MBq dosage in 0.2 ml saline into the tail vein. During the acquisitions, the animals were anaesthetised with 2% isoflurane in oxygen and placed on a dedicated bed (Mediso Ltd, Budapest, Hungary) in prone position. SPECT scans were performed in standard mode with 4 frames of 45 seconds each, 80 s time frame, for a total acquisition time of 1 hour, using a multi-pinhole collimator (nanoScan SPECT/CT system, Mediso Ltd., Budapest, Hungary) 30 min after radiotracer injection. Reconstructions were performed in Nucline software (Mediso Ltd., Budapest, Hungary) with the internal MHHQ image reconstruction parameter set applied, followed by manual segmentation of the respective brain areas. Obtained values were evaluated in comparison to brainstem using Fusion software (Mediso Ltd. Budapest, Hungary).

### RNA Sequencing and Analysis

A subset of animals was deeply anaesthetised with isoflurane (P8, N=8-10/ treatment group) or a mixture of ketamine (75 mg/kg) and xylazine (15mg/kg; adults, N= 5/ treatment group), followed by quick decapitation. Brains were manually removed 24 hours after PA induction or in adulthood under baseline conditions and immediately cooled on wet ice.  Brains were dissected into coronal blocks using a stainless steel brain mold. Bilateral medial prefrontal cortices were microdissected with a sterile blade, guided by the Paxinos and Watson atlas^26^. Total RNA was isolated from homogenized tissue samples using the RNeasy Lipid Tissue Mini Kit (Qiagen, UK). This was followed by DNase treatment to remove DNA contamination. The concentrations and quality of the extracted RNA samples were assessed using an Agilent TapeStation (Agilent Technologies, USA). All samples had a similar RNA Integrity Number (RIN) above 9.5. Libraries were prepared using the Illumina Stranded mRNA Prep, Ligation kit (Illumina, Inc., USA, #20040534) according to the manufacturer's protocol. Paired-end sequencing was performed on an Illumina NextSeq 2000 Sequencing System using NextSeq 2000 P3 Reagents (Illumina, Inc., USA, 20040560). FastQC (Babraham Bioinformatics, UK, Andrews, 2010, FastQC: a quality control tool for high throughput sequence data, https://www.bioinformatics.babraham.ac.uk/projects/fastqc/) was used to assess the quality of the raw sequence data. Alignment-independent gene-level quantifications were executed using the Salmon tool^27^ with the Rattus_norvegicus.mRatBN7.2.106.gtf annotation file from Ensembl BioMart. Subsequent analyses were performed using the R programming language (https://www.r-project.org/) and RStudio (version = 2023.6.1.524, <http://www.posit.co/>).

The tximport package (version = 1.24.0)^28^ was used to summarize the results into a data matrix. Gene expression analyses were performed using the DESeq2 package (version = 1.36.0)^29^ with pairwise contrasts for the investigated comparisons. Genes with at least 10 counts per group were considered relevant and retained for further investigations. To control for multiple testing, a false discovery rate (FDR) of 0.1 was applied for P8 samples and 0.2 for adult samples. Due to the prolonged moderate effects of the treatment, a more permissive FDR value was applied for the pathway analysis in adult samples. Volcano plots to visualize differential gene expression patterns were generated with the EnhancedVolcano R package (version = 1.14.0)^30^. Ranked lists were created from the log2 fold change (log2FC) and p-values of all experimentally investigated genes using signed p-values (rankings = sgn(log2FC * [-log10p-value]). Gene set enrichment analyses were conducted using the fgsea package (version=1.22.0)^31^ applying Normalized Enrichment Scores (NES), a statistical measure used to evaluate the degree to which a gene set is enriched at the top or bottom of a ranked list of genes. A positive NES value indicates that the gene set is enriched at the top of the ranked list, suggesting that the genes in this set are more highly expressed (positively correlated with the phenotype of interest), while a negative NES value indicates that the gene set is enriched at the bottom of the ranked list, suggesting that the genes in this set are more lowly expressed (negatively correlated with the phenotype of interest). The analyses utilized Gene Ontology: Biological Pathways and Gene Ontology: Molecular Functions gene set collections from the Molecular Signatures Database (MSigDB, <https://www.gsea-msigdb.org/gsea/msigdb/collections.jsp>). The analyses were performed using the fgseaMultilevel function, retrieving Benjamini-Hochberg correction-adjusted p-values for every pathway. Functional enrichment dotplots and pairwise similarity plots were generated using the clusterProfiler (version = 4.4.4) and enrichplot R packages (version = 1.16.2)^32^. On the dotplots gene sets with NES > 0 (“activated”) are enriched for genes with positive signed p-values (upregulated). In contrast, gene sets with NES < 0 (“suppressed”) are enriched for genes with negative signed p-values (downregulated) in the phenotype of interest. Pairwise similarity calculations are based on computing Jaccard similarity indices between all enriched terms using their gene sets (Jaccard = Number of shared genes between two terms/Total unique genes in both terms). The similarity matrix was used to perform hierarchical clustering using the hclust clustering method.

### Statistical analysis

Group allocation was performed by an experimenter not involved in data collection or analysis. During the experiments, investigators administering IL-1RA treatment and performing behavioural tests were blinded to group allocation. Outcome assessments, including histological, imaging, and molecular analyses, were conducted in a blinded manner, until all statistical tests were completed. Sample size determination was based on pilot data and prior studies of our lab, and previous publications using similar behavioural paradigms. Power analyses were conducted using G*Power 3.1.9.7, with an alpha level of 0.05, and a power (1–β) of 0.7, in line with commonly accepted standards. Behavioural data from males and females were collected and analysed independently, at no point were sexes pooled or mixed within groups for analysis. Behavioural testing and adult molecular/histological analyses were performed in independent cohorts to avoid confounding effects from prior testing. Animals were excluded if they exhibited signs of illness or if technical problems prevented reliable data collection. For behavioural testing, animals failing to engage with the task were excluded (e.g., one animal was excluded for unability to do nosepokes in the 5-CSRTT). Outlier data points (more than 2 SDs from the group mean) were assessed for technical errors and removed when justified (Supplementary table 7). For normality assessment GraphPad Prism (version 8.0.1, GraphPad Prism Software Inc., USA) and Python (version 3.10.0, https://www.python.org/) with *numpy* 1.26.4, *pandas* 1.5.3, and *scipy* 1.10.1 were used. In the case of synaptic boutons and microglia, outliers were excluded from visualisation using the ROUT method (Q = 5%).

# Supplementary figures and legends

**
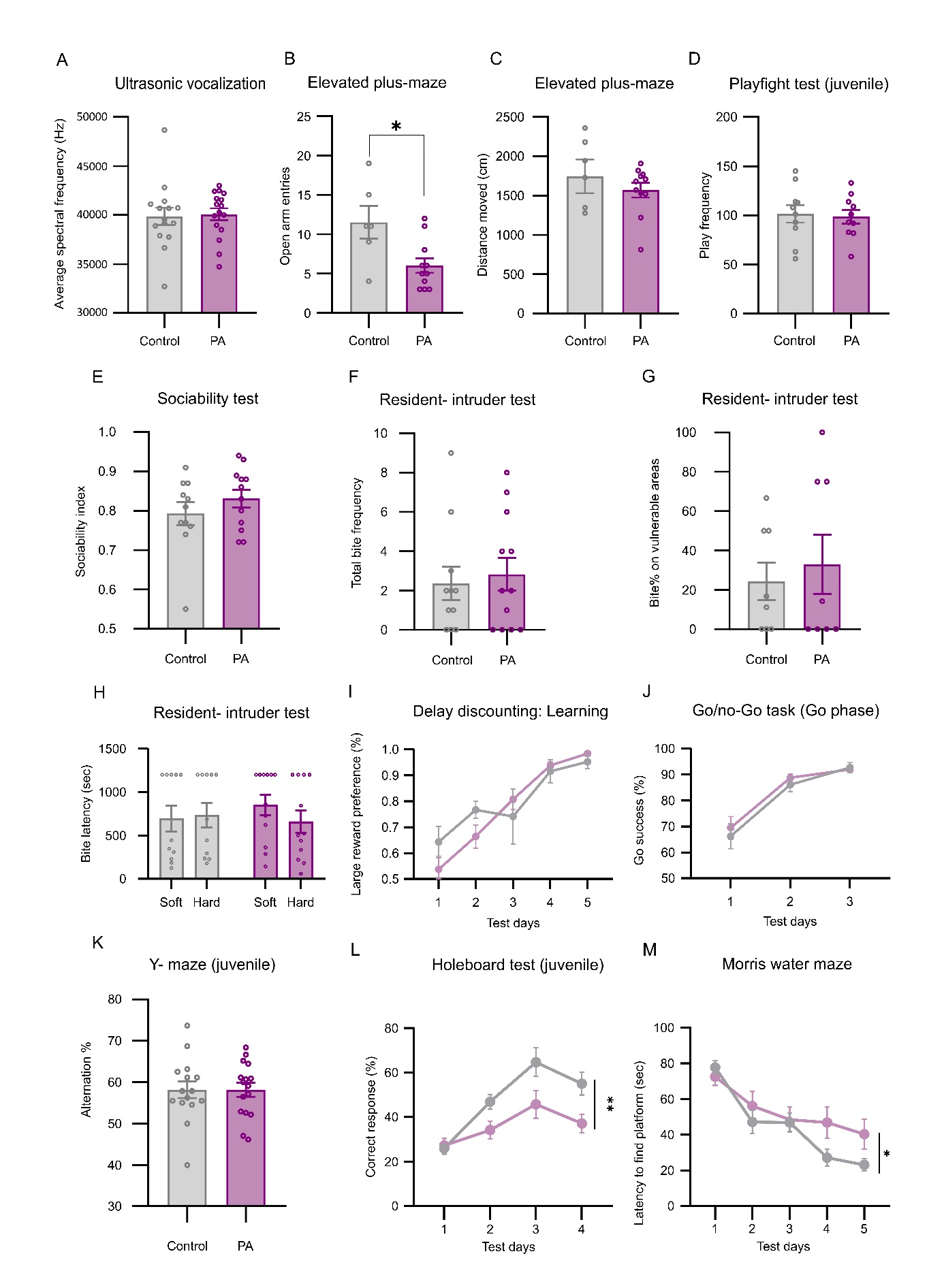
**

**Supplementary Figure 1 Long-Term Behavioural Consequences of the PA Insult in Males.** (**A**) PA animals showed no difference in ultrasonic vocalization spectral frequency compared to control. (**B-C**) PA animals showed decreased open arm frequency in the EPM (*t*(15)=2.771, *p=*0.014), but no difference in distance moved, suggesting an intact neuro-motor apparatus. (**D-H**) PA animals showed no apparent deficits during juvenile playfight test, and resident-intruder test. (**I**) During the learning phase of the delay discounting test, there was a significant increase in large reward preference (test day *F*(2.387, 35.81)= 31.65, *p<*0.001; treatment *F*(1, 15)= 2.31, *p=*0.149; test day × treatment interaction *F*(4, 60)= 0.842, *p=*0.5). (**J**) Experimental groups showed similar Go success% during the Go phase of the Go/no-Go task. (**K**) No differences were observed in the Y-maze. (**L-M**) PA animals showed decreased spatial learning abilities in the juvenile hole-board test (test day *F*(2.680, 77.71)=16.88, *p<* 0.0001; treatment *F*(1,29)=8.934, *p=* 0.005; test day × treatment interaction *F*(3,87)=2.546, *p=*0.061) and adult Morris water maze test (trial *F*(3.139, 62.79)= 30.77, *p<* 0.001; treatment *F*(1, 20)= 1.186, *p=* 0.289; trial × treatment interaction *F*(4, 80)= 2.954, *p=*0.024).

**
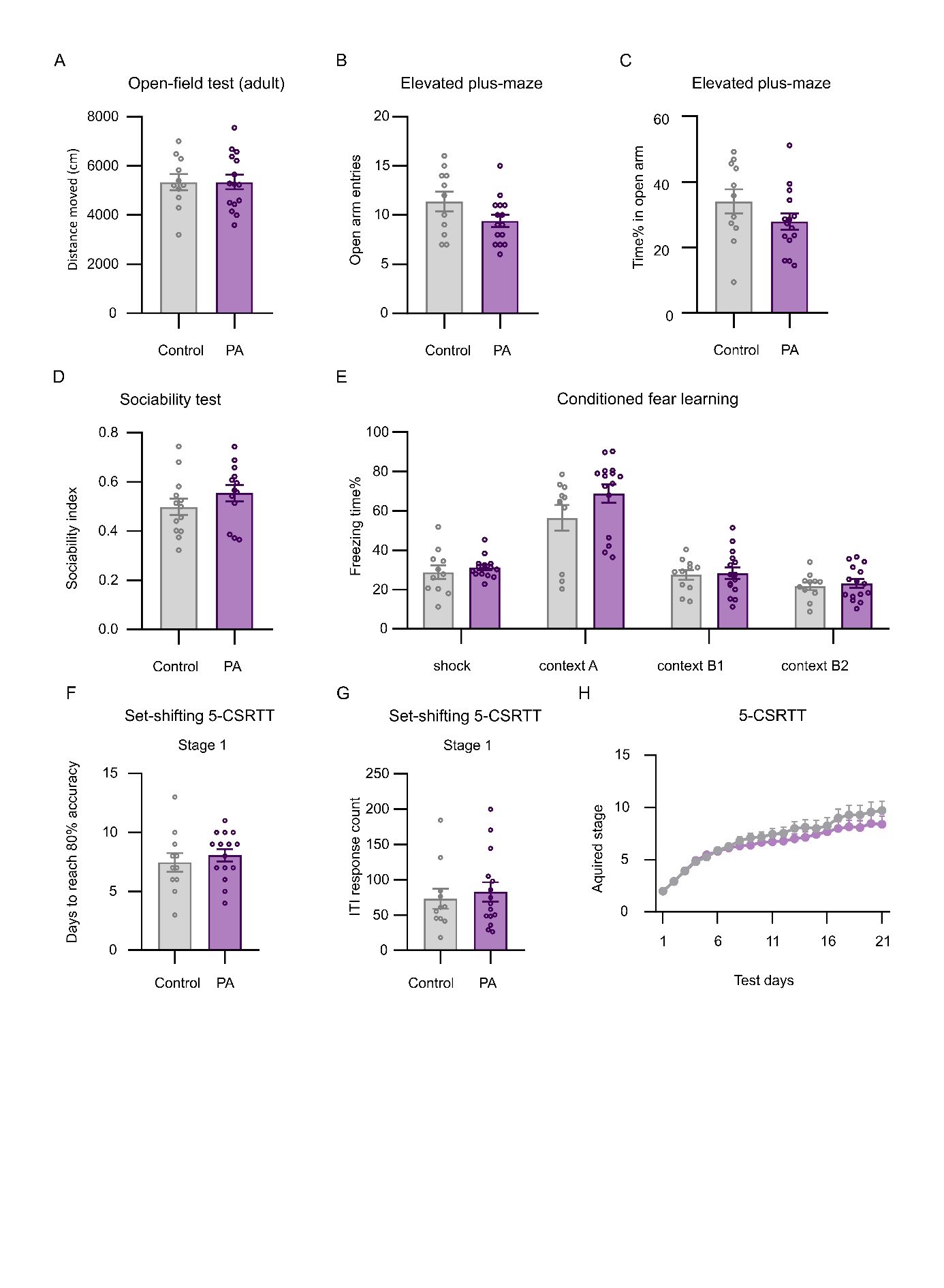
**

**Supplementary Figure 2 Long-Term Behavioural Consequences of the PA Insult in Females.** (**A**) No gross motor alterations were found during the open-field test in adulthood. (**B-C**) Anxiety testing in the elevated plus-maze performed in adulthood revealed no significant changes. (**D**) PA animals showed similar social interest in the sociability test. (**E**) PA did not affect conditioned fear learning and acquisition in females. (**F-H**) Female PA animals presented no learning and attention deficits and impulsivity in the set-shifted 5-choice serial reaction time task.


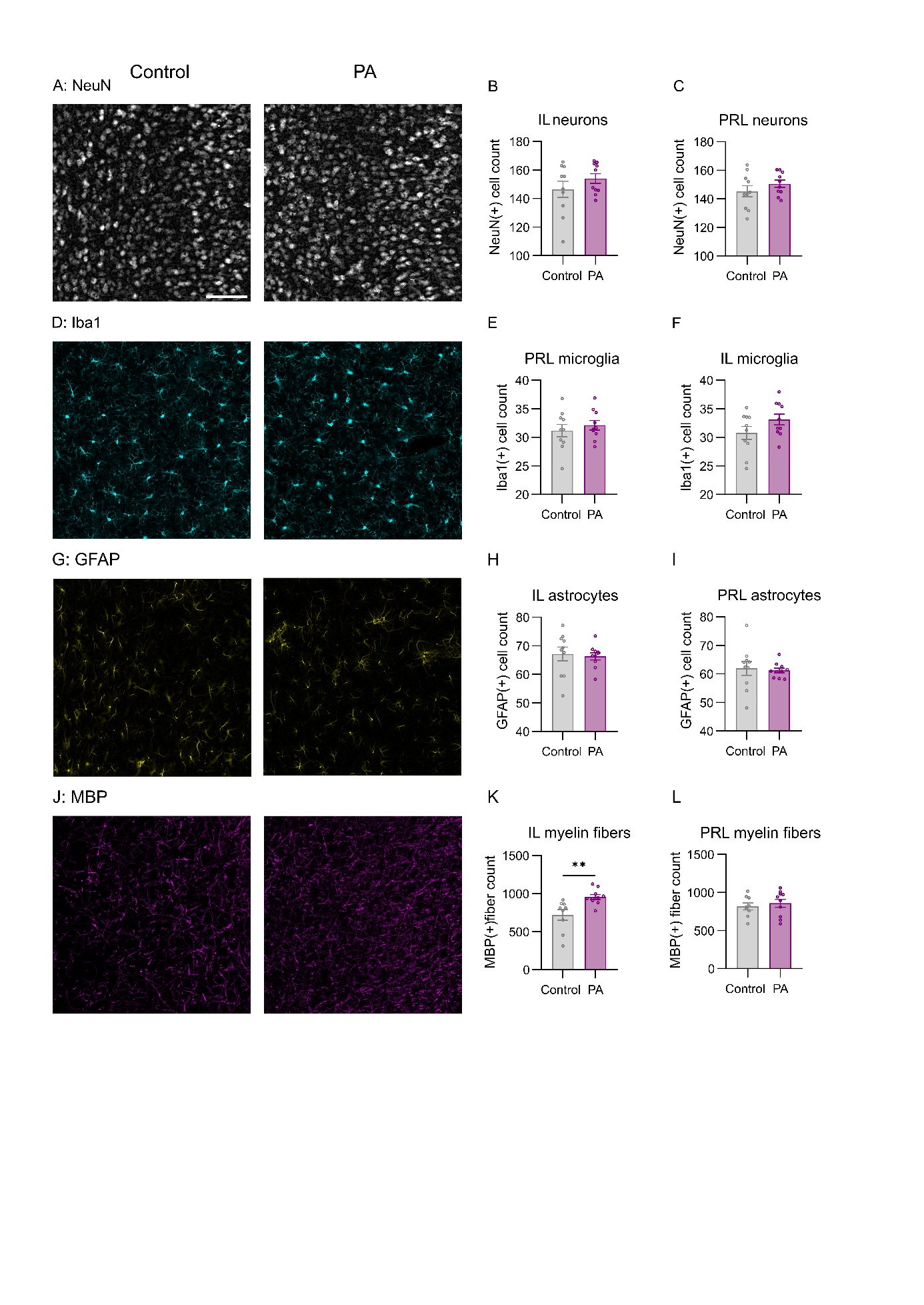


**Supplementary Figure 3 No Alterations in the Number of Neurons and Immune Cells in the IL and PRL of Adult Animals.** (**A-C**) Statistical comparisons of the areas of the investigated regions of interest (ROIs) and average NeuN(+) cell count for neurons in the infralimbic and prelimbic cortices reveal no significant differences between control and perinatal asphyxia (PA) animals in adulthood. Similarly, comparisons of Iba1(+) microglia (**D-F**) and GFAP(+) astrocytes (**G-I**) show no notable differences between groups. (**J-L**) Significant increase in the density of MBP(+) myelin fibres in the IL of PA animals (U= 8, *p=*0.001), paralleled by no alteration in the PRL.


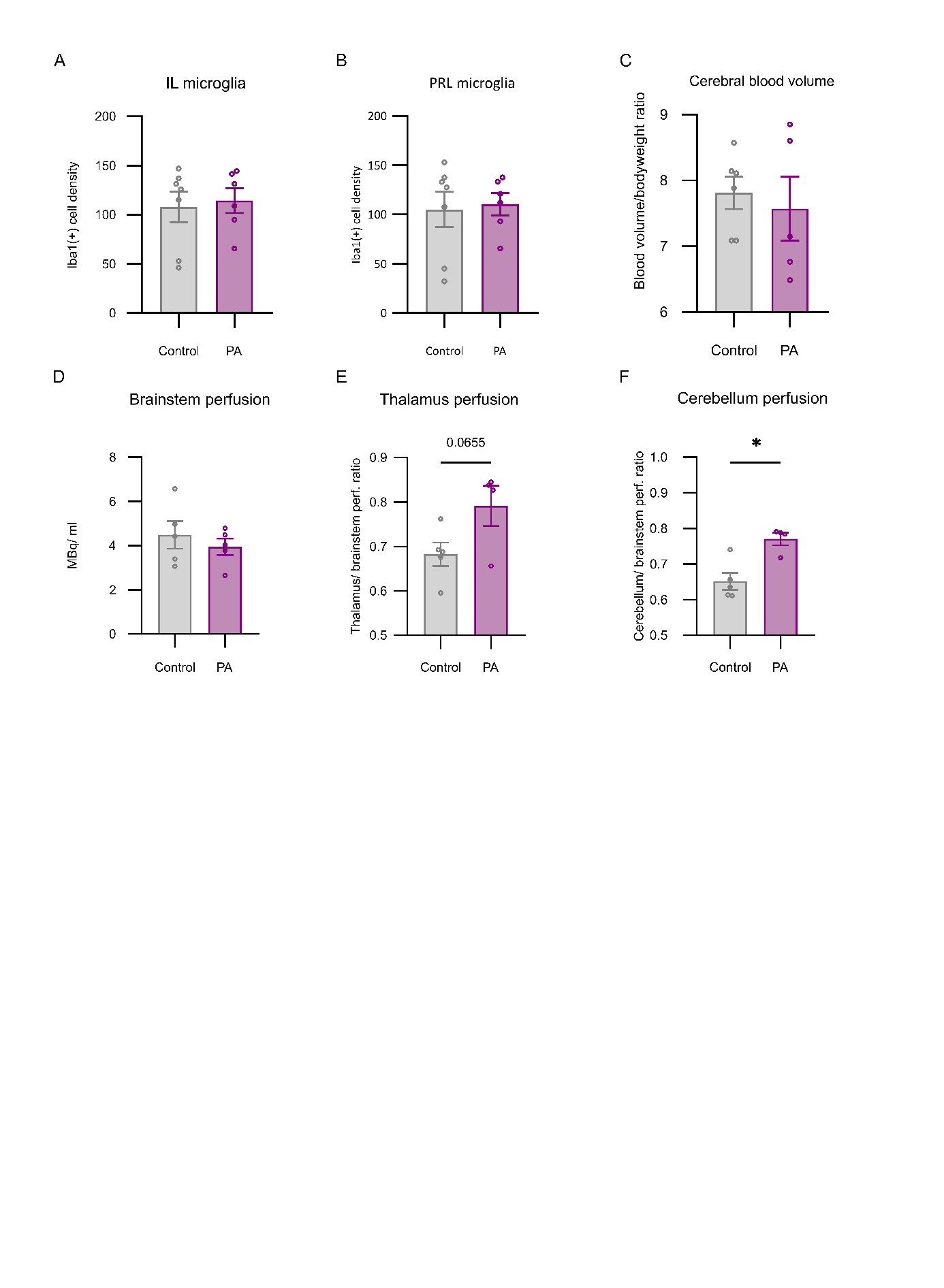


**Supplementary Figure 4 Acute Characterisation of PA Animals.** (**A-B**) Treatment groups showed no differences in mPFC microglia densities 24 hours after the PA insult. (**C**) MRI imaging 24 hours after PA demonstrated similar brain blood volumes in ratio to bodyweight in control and PA animals. (**D-F**) During acute SPECT imaging, brainstem perfusion remained on a control level. Local relative perfusion changes were apparent in the thalamus (*t*(7)= 2.182, *p=*0.065) and cerebellum (U=1, *p=*0.031) of PA animals.


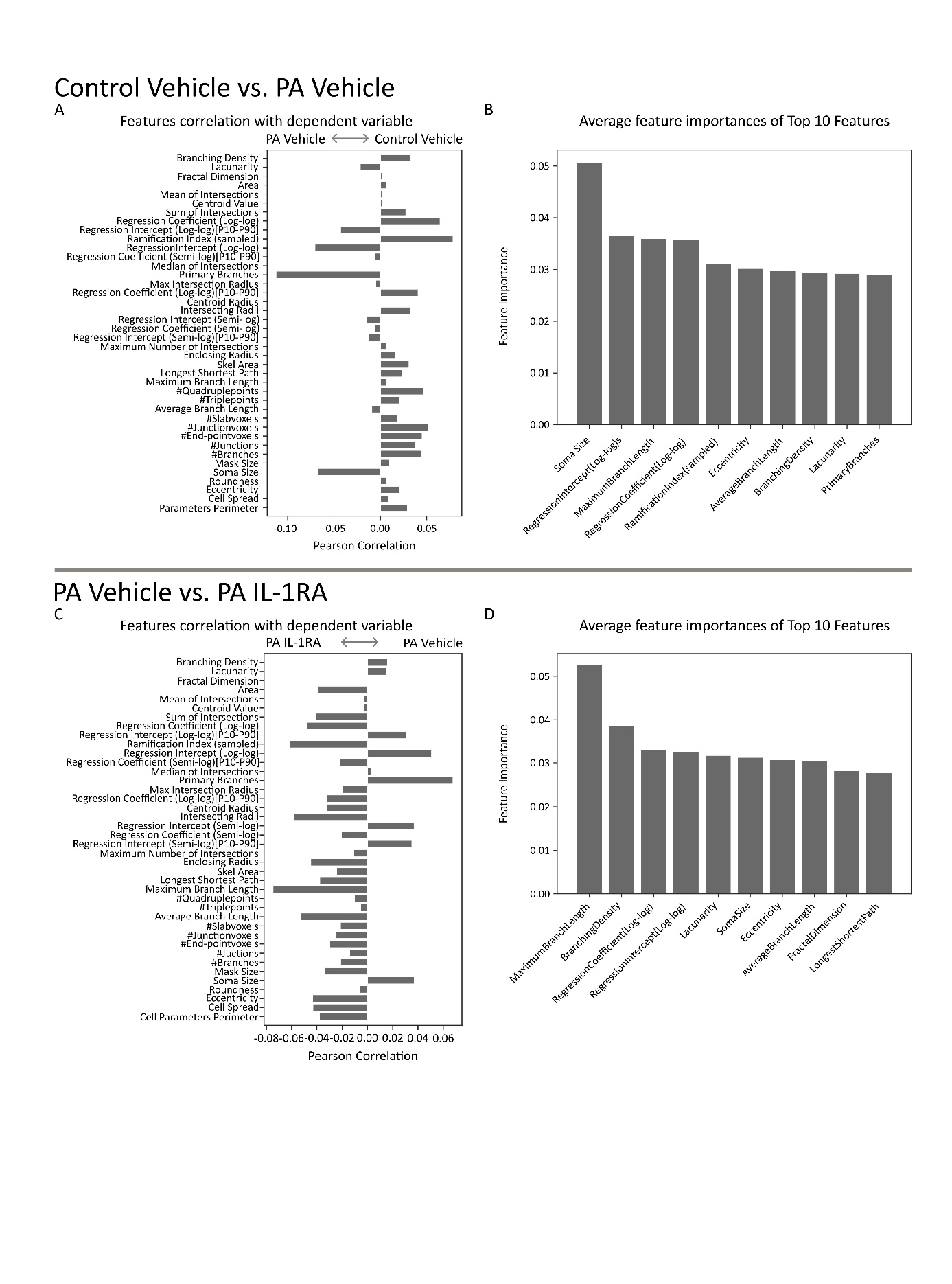


**Supplementary Figure 5 Comparison of Feature Importances in Predicting Microglial Phenotypes.** (**A**) Pearson correlation analysis revealed that the number of primary branches, the regression intercept (log-log scale), and soma sizes exhibited the strongest correlations with the PA phenotype. (**B**) In contrast, a Random Forest classifier identified soma sizes, maximum branch lengths, and the radii of the maximum intersections as the most important factors for distinguishing PA and control microglia. (**C**) In the rescue experiment using IL-1RA after PA insults, average branch lengths, regression coefficients, and the number of intersecting radii showed the most significant changes. (**D**) Lacunarity, maximum branch lengths, and radii of the maximum intersections were the top discriminators between PA IL-1RA and PA vehicle animals.


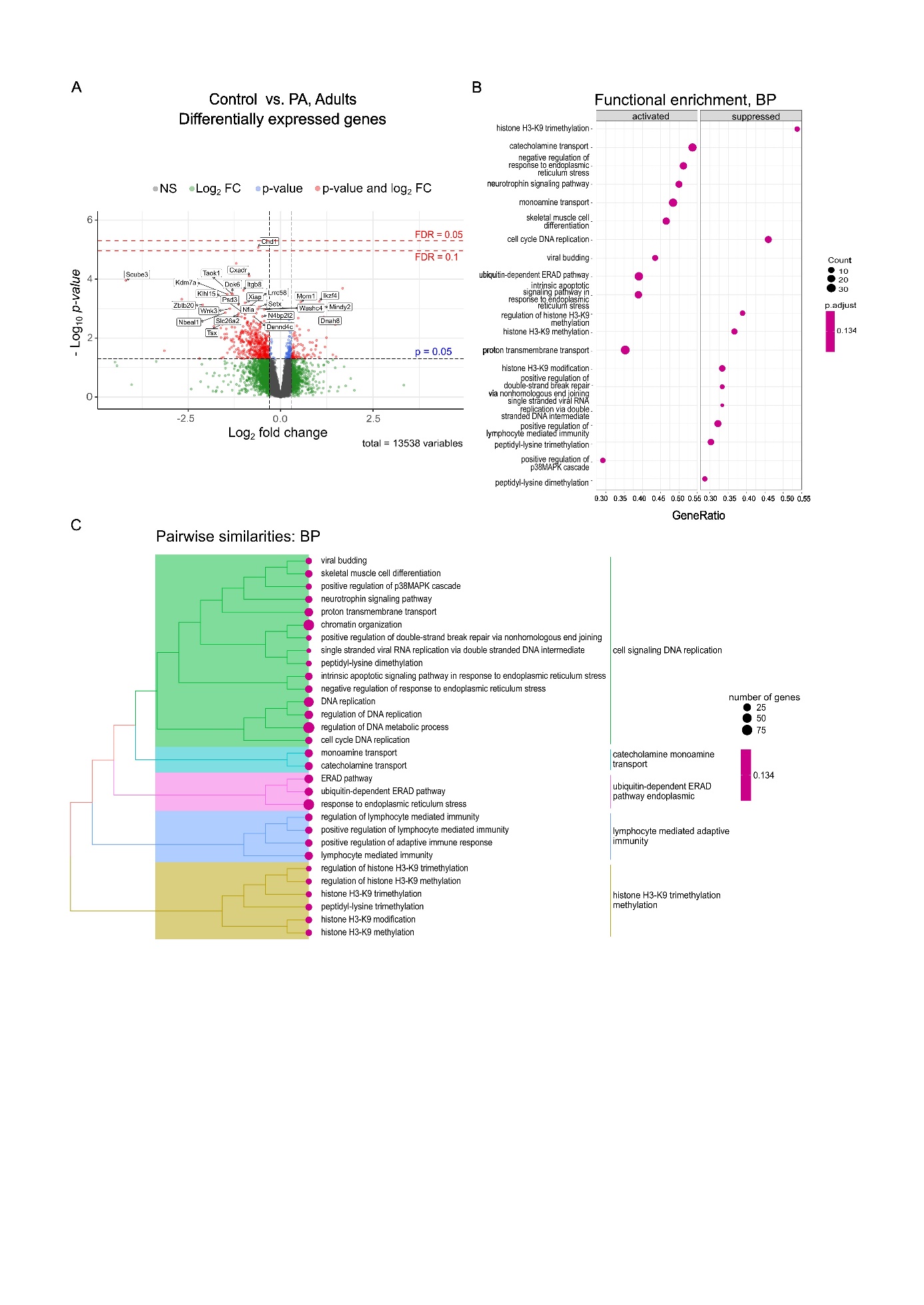


**Supplementary Figure 6 Long-term Molecular and Pathway Alterations following PA in the mPFC of males** (A) Experiment 1: Differential gene expression analysis of bulk medial prefrontal cortex (mPFC) samples isolated 6 months after PA in baseline conditions revealed moderate differences between treatment groups, with 1 gene reaching an FDR < 0.1 and 614 genes showing nominal significance (p < 0.05) out of 13,538 tested. (B-C) Functional gene set enrichment analysis using Gene Ontology (GO) Biological Processes and hierarchical clustering of pairwise similarities among altered pathways based on shared genes, identifies dominant clusters related to epigenetic regulation (particularily histone mondification), as well as immune responsivity and cell signaling pathways.

**Supplementary Table 1.** Primary antibodies used in this study.

| **Reagent** | **Source** | **Catalogue no.** | **Host** | **Dilution** |
| --- | --- | --- | --- | --- |
| CB1 | Immunogenes | 03 | Rabbit | 1:3000 |
| GFAP | Sigma | G3893 | Mouse | 1:3000 |
| IBA1 | Wako | 019-19741 | Rabbit | 1:10000 |
| Kv.2.1 | NeuroMab | 75-014 | Mouse | 1:1000 |
| MBP | Abcam | ab7349 | Rat | 1:500 |
| NeuN | Synaptic Systems | 266006 | Chicken | 1:1000 |
| PV | Swant | PVG213 | Goat | 1:5000 |
| VGAT | Synaptic Systems | 131003 | Rabbit | 1:900 |
| VGAT | Synaptic Systems | 131004 | Guinea-pig | 1:1000 |
| VGLUT1 | Synaptic Systems | 135304 | Guinea-pig | 1:2000 |
| VGLUT2 | Synaptic Systems | 135416 | Chicken | 1:900 |

**Supplementary Table 2.** Secondary antibodies and fluorescent dyes used in this study.

| **Reagent** | **Source** | **Catalogue no.** | **Host** | **Dilution** |
| --- | --- | --- | --- | --- |
| Alexa Fluor Plus 405 anti-rabbit IgG | Invitrogen | A48258 | Donkey | 1:500 |
| Alexa Fluor 488 anti-rabbit IgG | Invitrogen | A-21206 | Donkey | 1:500 |
| Alexa Fluor 488 anti-rabbit IgG | Jackson ImmunoResearch | 111-545-144 | Goat | 1:500 |
| Alexa Fluor 488 anti-goat IgG | Invitrogen | A-11055 | Donkey | 1:500 |
| Alexa Fluor 568 anti-guinea pig IgG | Invitrogen | A-11075 | Goat | 1:500 |
| Cy3 anti-mouse IgG | Jackson ImmunoResearch | 715-165-151 | Donkey | 1:500 |
| Cy3 anti-guinea pig IgG | Jackson  ImmunoResearch | 706-165-148 | Donkey | 1:500 |
| Alexa Fluor 647 anti-chicken IgG | Invitrogen | A78952 | Donkey | 1:500 |
| Alexa Fluor 647 anti-chicken IgY | Jackson  ImmunoResearch | 703-605-155 | Donkey | 1:500 |
| Alexa Fluor 647 anti-rat IgG | Invitrogen | A78947 | Donkey | 1:500 |
| Alexa Fluor 647 anti-mouse IgG | Jackson  ImmunoResearch | 715-605-151 | Donkey | 1:500 |

**Supplementary table 3.** Top Differentially Expressed Genes for Control vs. Perinatal Asphyxia Comparisons

| **Gene symbol** | **baseMean** | **log2FoldChange** | **p value** | **Adjusted p value** |
| --- | --- | --- | --- | --- |
| Camk4 | 627.545 | 0.387 | 7.52E-07 | 5.32E-03 |
| Dbp | 461.65 | -0.44 | 6.22E-07 | 5.32E-03 |
| Igsf9 | 706.118 | -0.408 | 5.91E-06 | 0.028 |
| Col24a1 | 193.81 | 0.428 | 1.05E-05 | 0.037 |
| RGD1307929 | 5647.014 | 0.147 | 3.98E-05 | 0.113 |
| Gpc2 | 221.304 | -0.557 | 1.74E-04 | 0.247 |
| Arhgef3 | 466.952 | 0.302 | 1.40E-04 | 0.247 |
| Mapk1ip1 | 635.372 | -0.164 | 1.58E-04 | 0.247 |
| Acss2 | 1154.19 | -0.178 | 1.20E-04 | 0.247 |
| Cap2 | 792.51 | 0.349 | 1.30E-04 | 0.247 |
| Rasl11a | 115.079 | 0.413 | 4.14E-04 | 0.308 |
| Rgs4 | 9610.192 | 0.244 | 6.09E-04 | 0.308 |
| Tuba4a | 4074.18 | 0.238 | 4.35E-04 | 0.308 |
| Pttg1 | 44.712 | 0.593 | 4.45E-04 | 0.308 |
| Cpd | 540.11 | 0.313 | 6.07E-04 | 0.308 |
| Kcnv1 | 1335.48 | 0.276 | 4.85E-04 | 0.308 |
| Kcnq3 | 921.171 | 0.227 | 3.68E-04 | 0.308 |
| Rps6kl1 | 993.86 | -0.217 | 2.45E-04 | 0.308 |
| Tle2 | 308.736 | -0.259 | 5.24E-04 | 0.308 |
| Rab33a | 446.15 | -0.188 | 2.62E-04 | 0.308 |

The table summarizes the top 20 differentially expressed genes in the medial prefrontal cortex (mPFC) when comparing the control vehicle group to the PA vehicle group. The table presents the mean RNA-seq raw read counts for each gene in the control vehicle group (baseMean) and the log2 fold change values (log2FoldChange) in the PA vehicle group. Statistical significance was determined using the Wald test for p-values, and the Benjamini-Hochberg (BH) method was applied to calculate adjusted p-values (p adj), controlling the false discovery rate (FDR) and correcting for multiple testing.

**Supplementary Table 4.** Sample sizes for behavioural tests

| **Cohort ID** | **Animals/ treatment group** | **Sex** | **Name and order of tests performed** |
| --- | --- | --- | --- |
| Cohort 1 | 6-11 | male | Adult behavioural tests (open field test, elevated plus maze test, delay discounting test) |
| Cohort 2 | 11-12 | male | Adult behavioural tests (rotarod, Morris water maze, social recognition, resident-intruder test) |
| Cohort 3 | 7-9 | male | Adult behavioural tests (social interaction, 5-choice serial reaction task, go/no-go test) |
| Cohort 4 | 15-16 | male | Acute neuromotor testing and juvenile tests (juvenile open field test, playfight test, Y-maze, holeboard test) |
| Cohort 5 | 5-5 | male | Adult immunohistochemistry |
| Cohort 6 | 7-8 | male | Acute hormone measurements (number/ timepoint) |
| Cohort 7 | 6-7 | male | Acute immunohistochemistry |
| Cohort 8 | 9-11 | male | Acute imaging studies |
| Cohort 9 | 12-15 | male | IL-1RA treatment, adult behavioural tests  (EPM, 5-choice serial reaction time test) |
| Cohort 10 | 5-6 | male | IL-1RA treatment, adult immunohistochemistry |
| Cohort 11 | 7-9 | male | IL-1RA treatment, acute immunohistochemistry |
| Cohort 12 | 8-10 | male | IL-1RA treatment, acute RNAseq analysis |
| Cohort 13 | 11-15 | female | Adult behavioural tests (open field test, 5-choice serial reaction task) |
| Cohort 14 | 11-15 | female | Adult behavioural tests (elevated plus maze, sociability, conditioned fear learning) |

**Supplementary Table 5.** Assessment of normality and variance assumptions for behavioural analysis

|  |  | **Control** | | | **ASX** | | | **All groups** | | |  |
| --- | --- | --- | --- | --- | --- | --- | --- | --- | --- | --- | --- |
|  |  | **Shapiro-Wilk test** | | | | | | **Brown-Forsythe test** | | |  |
| **Males** | **Behavioral variable** | **Normally distributed** | **F-value** | **p-value** | **Normally distributed** | **F-value** | **p-value** | **Significant** | **F-value** | **p-value** | **Statistical test performed** |
|  | Elevated plus maze distance moved | Yes | 0.951 | 0.754 | No | 0.843 | 0.035 | No | 2.36 | 0.145 | Mann-Whitney U test |
|  | Elevated plus maze open arm entries | Yes | 0.975 | 0.929 | Yes | 0.859 | 0.057 | No | 0.78 | 0.389 | Unpaired t-test |
|  | Elevated plus maze open arm% | Yes | 0.980 | 0.954 | No | 0.799 | 0.014 | No | 1.77 | 0.203 | Mann-Whitney U test |
|  | Negative geotaxis test | Yes | 0.934 | 0.319 | No | 0.826 | 0.006 | Yes | 6.82 | 0.014 | Mann-Whitney U test |
|  | Open field test | Yes | 0.815 | 0.08 | Yes | 0.886 | 0.127 | No | 0.347 | 0.564 | Unpaired t-test |
|  | Playfight test | Yes | 0.962 | 0.814 | Yes | 0.984 | 0.984 | No | 0.462 | 0.505 | Unpaired t-test |
|  | Resident-intruder test bite% on vulnerable parts | No | 0.750 | 0.002 | No | 0.807 | 0.011 | No | 0.515 | 0.48 | Mann-Whitney U test |
|  | Resident-intruder test hard bite latency | No | 0.769 | 0.003 | No | 0.858 | 0.046 | No | 0.292 | 0.594 | Mann-Whitney U test |
|  | Resident-intruder test soft bite latency | Yes | 0.83 | 0.06 | No | 0.757 | 0.009 | No | 0.637 | 0.438 | Mann-Whitney U test |
|  | Resident-intruder test total bite frequency | No | 0.798 | 0.009 | Yes | 0.87 | 0.065 | No | 0.398 | 0.534 | Mann-Whitney U test |
|  | Righting reflex test | Yes | 0.924 | 0.228 | Yes | 0.907 | 0.107 | No | 0.376 | 0.544 | Unpaired t-test |
|  | Rotarod test | Yes | 0.9 | 0.189 | Yes | 0.909 | 0.212 | No | 0.703 | 0.41 | Unpaired t-test |
|  | Sociability test | Yes | 0.863 | 0.063 | Yes | 0.925 | 0.334 | No | 0.004 | 0.948 | Mann-Whitney U test |
|  | Social interaction test | Yes | 0.858 | 0.092 | No | 0.807 | 0.048 | No | 0.006 | 0.937 | Mann-Whitney U test |
|  | USV average spectral frequency | Yes | 0.91 | 0.135 | Yes | 0.917 | 0.154 | No | 0.308 | 0.582 | Unpaired t-test |
|  | USV frequency | No | 0.844 | 0.014 | Yes | 0.948 | 0.467 | No | 0.339 | 0.564 | Mann-Whitney U test |
|  | Y-maze | Yes | 0.948 | 0.506 | Yes | 0.959 | 0.652 | No | 0.011 | 0.916 | Unpaired t-test |
|  | Elevated plus maze (IL-1RA treatment) | Yes (vehicle); Yes (IL-1RA) | 0.861 (vehicle); 0.892 (IL-1RA) | 0.051(vehicle); 0.127 (IL-1RA) | Yes (vehicle); Yes (IL-1RA) | 0.96 (vehicle); 0.922 (IL-1RA) | 0.805 (vehicle); 0.211(IL1-RA) | No | 0.526 | 0.666 | Two-way ANOVA |
| **Females** | Elevated plus maze frequency | Yes | 0.916 | 0.29 | Yes | 0.942 | 0.418 | No | 2.49 | 0.127 | Unpaired t-test |
|  | Elevated plus maze time% | Yes | 0.939 | 0.51 | Yes | 0.942 | 0.409 | No | 1.198 | 0.284 | Unpaired t-test |
|  | Open field | Yes | 0.976 | 0.942 | Yes | 0.962 | 0.742 | No | 0.304 | 0.585 | Unpaired t-test |
|  | Set-shifted 5-CSRTT phase 1 days to reach 80% | Yes | 0.972 | 0.907 | Yes | 0.938 | 0.363 | No | 0.225 | 0.639 | Unpaired t-test |
|  | Set-shifted 5-CSRTT phase 1 ITI responses | No | 0.845 | 0.037 | No | 0.881 | 0.049 | No | 0.329 | 0.571 | Mann-Whitney U tests |
|  | Sociability test | Yes | 0.958 | 0.724 | Yes | 0.93 | 0.348 | No | 0.001 | 0.968 | Unpaired t-test |

**Supplementary Table 6.** Evaluation for the assumpion of sphericity in behavioral tests involving repeated measures

| **Behavioral test** | **Sphericity** | **Original degrees of freedom** | **Corrected degrees of freedom** | **Corrected Time Effect** | **Corrected Treatment Effect** |
| --- | --- | --- | --- | --- | --- |
| 5-CSRTT, males | GG epsilon: 1.0000  HF epsilon: 0.0345 | Condition df: 29; Error df: 348 | GG - Condition df: 29.00, Error df: 348.00  HF- Condition df: 1.00, Error df: 12.00 | F(29, 348)= 28.7443, p<0.0001;  GG corrected p< 0.0001  HF corrected p<0.0002 | F(29, 348)= 33.1092; p< 0.0001 |
| Delay discounting | GG epsilon: 1.0000  HF epsilon: 1.0000 | Condition df: 7; Error df: 112 | GG - Condition df: 7.00, Error df: 112.00  HF - Condition df: 7.00, Error df: 112.00 | F(7, 112)= 10.2655, p<0.0001;  GG corrected p< 0.0001  HF corrected p<0.0001 | F(7, 112)=6.0536;  p= 0.0152 |
| Delay discountin learning | GG epsilon: 1.0000  HF epsilon: 1.0000 | Condition df: 4; Error df: 64 | GG- Condition df: 4; Error df: 64 HF- Condition df: 4; Error df: 64 | F(4, 64)= 26.00064, p<0.0001;  GG corrected p< 0.0001  HF corrected p<0.0001 | F(4, 64)= 2.1249; p= 0.1487 |
| Go/NoGo task, Go success | GG epsilon: 0.5000  HF epsilon: 0.5000 | Condition df: 2; Error df: 20 | GG- Condition df: 1; Error df: 10 HF- Condition df: 1; Error df: 10 | F(2, 20)= 76.1064, p<0.0001;  GG corrected p< 0.0001  HF corrected p<0.0001 | F(2, 20)=0.1889; p=0.668 |
| Go/NoGo task, total correct Go | GG epsilon: 1.0000  HF epsilon: 1.0000 | Condition df: 12; Error df: 168 | GG- Condition df: 12; Error df: 168 HF- Condition df: 12; Error df: 168 | F(12, 168)= 0.2278, p=0.9969;  GG corrected p=0.9968  HF corrected p=0.9968 | F(12, 168)=3.0202; p=0.0838 |
| Go/NoGo task, total correct NoGo | GG epsilon: 1.0000  HF epsilon: 1.0000 | Condition df: 12; Error df: 156 | GG- Condition df: 12; Error df: 156 HF- Condition df: 12; Error df: 156 | F(12, 156)=7.9483, p=<0.0001;  GG corrected p<0.0001;  HF corrected p<0.0001 | F(12, 156)=25.7612;  p<0.0001 |
| Hole board test | GG epsilon: 1.0000  HF epsilon: 1.0000 | Condition df: 3; Error df: 90 | GG- Condition df: 3; Error df: 90 HF- Condition df: 3; Error df: 90 | F(3, 90)=11.8247, p<0.0001;  GG corrected p< 0.0001  HF corrected p<0.0001 | F(3, 90)=7.7240; p=0.0063 |
| Morris water maze test | GG epsilon: 1.0000  HF epsilon: 1.0000 | Condition df: 4; Error df: 84 | GG- Condition df: 4; Error df: 84 HF- Condition df: 4; Error df: 84 | F(4, 84)=11.7821, p<0.0001;  GG corrected p< 0.0001  HF corrected p<0.0001 | F(4, 84)=2.7850; p=0.0980 |
| 5-CSRTT (IL-1RA treatment) | GG epsilon: 1.0000  HF epsilon: 1.0000 | Condition df: 29; Error df: 1363 | GG- Condition df: 29; Error df: 1363 HF- Condition df: 29; Error df: 1363 | F(29, 1363)=61.8247, p<0.0001;  GG corrected p< 0.0001  HF corrected p<0.0001 | F(29, 1363)=28.3481  p<0.0001 |
| Set-shifted 5-CSRTT phase 2 | GG epsilon: 1.0000  HF epsilon: 1.0000 | Condition df: 11; Error df: 275 | GG- Condition df: 11; Error df: 275 HF- Condition df: 11; Error df: 275 | F(11, 275)=63,1748, p<0.0001;  GG corrected p< 0.0001  HF corrected p<0.0001 | F(11, 275)=1.2951  p=0.2560 |

**Supplementary Table 7.** Justification for outlier removal

| **Test** | **Number and treatment group of outliers** | **Justification** |
| --- | --- | --- |
| Juvenile Open field | 1, control | Video artefact |
| 5-choice serial reaction time task | 1, control | Inability to learn nose poking |
| Histological analysis | 1, control; 1, PA | Brain perfusion error (visible blood in cerebral vessels) |
| Removal from experiment | 1 litter of animals | Low average weight and small litter size at weaning |

**Supplementary Table 8.** Summary table of effect size estimates

| **Statistical test** | **Behavioral test** | **Basis for effect size calculation** | **Effect size (f or d)** | **Alpha** | **Power** | **Estimated Sample Size (per group)** | **Used Sample Size (per group, males/ females)** |
| --- | --- | --- | --- | --- | --- | --- | --- |
| Mann-Whitney U test | Elevated plus maze | Previous studies showing robust effects on open arm entries^33^ | 1.3 | 0.05 | 0.7 | 8 | 6-11 / 11-15 |
| Mann-Whitney U test | Social interaction test, sociability test | Pilot studies and literature data reporting large effect sizes for behavioral changes in sniffing and following^34^ | 1.3 | 0.05 | 0.7 | 8 | 6-11/ 11-15 |
| Unpaired t-test; Mann-Whitney U test | Righting reflex test, ultrasound vocalization test | Literature data reporting large effect sizes in motor impairment studies^35^ and significant alterations in pup ultrasonic vocalization following early-life stress^36^ | 1 | 0.05 | 0.7 | 14 | 15-16 |
| Mann-Whitney U test | Resident-intruder test | Previous studies showing robust effects on aggressive behavior^37^ | 1.2 | 0.05 | 0.7 | 10 | 11-12 |
| Unpaired t-test | Playfight test | Literature data reporting large effect size peripubertal stress study^38^ | 1.2 | 0.05 | 0.7 | 10 | 10-10 |
| Unpaired t-test | Open field test | Previous studies showing robust effects on centrum time^33^ | 1.2 | 0.05 | 0.7 | 10 | 6-11/ 11-15 |
| Unpaired t-test | Y-maze test | Literature data reporting large effect size reflecting working memory impairment and hyperactivity^39,40^ | 1.2 | 0.05 | 0.7 | 10 | 15-16 |
| Unpaired t-test | Rotarod | Literature data reporting large effect size in brain injury models^40^ | 1.2 | 0.05 | 0.7 | 10 | 11-12 |
| Unpaired t-test | Conditioned fear learning | Previous studies showing robust effects on fear learning and generalization^41^ | 1 | 0.05 | 0.7 | 14 | 11-15 |
| Repeated measures ANOVA | Morris water maze test | Pilot studies showing robust effects on spatial learning and memory | 1 | 0.05 | 0.7 | 8 | 10-12 |
| Repeated measures ANOVA | Holeboard learning test | Literature data reporting large effect size in post-ischaemia rats^42^ | 1 | 0.05 | 0.7 | 8 | 15-16 |
| Repeated measures ANOVA | Delay Discounting task | Literature data reporting large effect size with stable trait-like performance^43,44^ | 1 | 0.05 | 0.7 | 8 | 6-11 |
| Repeated measures ANOVA | 5-choice serial reaction time task, set-shifting 5-choice serial reaction time task | Previous studies showing robust and consistent effects on attention and operant learning^41^ | 1 | 0.05 | 0.7 | 8 | 7-8/ 11-15 |
| Repeated measures ANOVA | Go/ no-Go task | Literature reporting a significant correlation between symptoms of impulsivity with no-go errors^45^ | 1 | 0.05 | 0.7 | 8 | 7-9 |

### References

1. Altman J, Sudarshan K. Postnatal development of locomotion in the laboratory rat. *Anim Behav*. 1975;23(4):896-920. doi:10.1016/0003-3472(75)90114-1

2. Horiquini Barbosa E, Vallim JH, Lachat J-J, de Castro VLSS. Assessments of Motor Abnormalities on the Grid-Walking and Foot-Fault Tests From Undernutrition in Wistar Rats. *J Mot Behav*. 2016;48(1):5-12. doi:10.1080/00222895.2015.1024824

3. Hofer MA. Multiple regulators of ultrasonic vocalization in the infant rat. *Psychoneuroendocrinology*. 1996;21(2):203-217. doi:10.1016/0306-4530(95)00042-9

4. Török B, Fodor A, Zsebők S, Sipos E, Zelena D. The Effect of Vasopressin Antagonists on Maternal-Separation-Induced Ultrasonic Vocalization and Stress-Hormone Level Increase during the Early Postnatal Period. *Brain Sci*. 2021;11(4). doi:10.3390/brainsci11040444

5. Vanderschuren LJ, Niesink RJ, Van Ree JM. The neurobiology of social play behavior in rats. *Neurosci Biobehav Rev*. 1997;21(3):309-326. doi:10.1016/s0149-7634(96)00020-6

6. Veenema AH, Neumann ID. Maternal separation enhances offensive play-fighting, basal corticosterone and hypothalamic vasopressin mRNA expression in juvenile male rats. *Psychoneuroendocrinology*. 2009;34(3):463-467. doi:10.1016/j.psyneuen.2008.10.017

7. Vorhees C V, Williams MT. Assessing spatial learning and memory in rodents. *ILAR J*. 2014;55(2):310-332. doi:10.1093/ilar/ilu013

8. Kraeuter A-K, Guest PC, Sarnyai Z. The Y-Maze for Assessment of Spatial Working and Reference Memory in Mice. *Methods Mol Biol*. 2019;1916:105-111. doi:10.1007/978-1-4939-8994-2_10

9. Shiotsuki H, Yoshimi K, Shimo Y, et al. A rotarod test for evaluation of motor skill learning. *J Neurosci Methods*. 2010;189(2):180-185. doi:10.1016/j.jneumeth.2010.03.026

10. Haller J, Nagy R, Toth M, Pelczer KG, Mikics E. NR2B subunit-specific NMDA antagonist Ro25-6981 inhibits the expression of conditioned fear: a comparison with the NMDA antagonist MK-801 and fluoxetine. *Behav Pharmacol*. 2011;22(2):113-121. doi:10.1097/FBP.0b013e328343d7b2

11. Pellow S, Chopin P, File SE, Briley M. Validation of open:closed arm entries in an elevated plus-maze as a measure of anxiety in the rat. *J Neurosci Methods*. 1985;14(3):149-167. doi:10.1016/0165-0270(85)90031-7

12. Adriani W, Seta D Della, Dessì-Fulgheri F, Farabollini F, Laviola G. Altered profiles of spontaneous novelty seeking, impulsive behavior, and response to D-amphetamine in rats perinatally exposed to bisphenol A. *Environ Health Perspect*. 2003;111(4). doi:10.1289/ehp.5856

13. Morris R. Developments of a water-maze procedure for studying spatial learning in the rat. *J Neurosci Methods*. 1984;11(1):47-60. doi:10.1016/0165-0270(84)90007-4

14. Bari A, Dalley JW, Robbins TW. The application of the 5-choice serial reaction time task for the assessment of visual attentional processes and impulse control in rats. *Nat Protoc*. 2008;3(5):759-767. doi:10.1038/nprot.2008.41

15. Winstanley CA, Eagle DM, Robbins TW. Behavioral models of impulsivity in relation to ADHD: translation between clinical and preclinical studies. *Clin Psychol Rev*. 2006;26(4):379-395. doi:10.1016/j.cpr.2006.01.001

16. File SE, Hyde JR. Can social interaction be used to measure anxiety? *Br J Pharmacol*. 1978;62(1):19-24. doi:10.1111/j.1476-5381.1978.tb07001.x

17. Deiana S, Watanabe A, Yamasaki Y, et al. MK-801-induced deficits in social recognition in rats: Reversal by aripiprazole, but not olanzapine, risperidone, or cannabidiol. *Behav Pharmacol*. 2015;26(8):748-765. doi:10.1097/FBP.0000000000000178

18. Koolhaas JM, Coppens CM, de Boer SF, Buwalda B, Meerlo P, Timmermans PJA. The resident-intruder paradigm: a standardized test for aggression, violence and social stress. *J Vis Exp*. 2013;(77):e4367. doi:10.3791/4367

19. Szente L, Balla GY, Varga ZK, et al. Endocannabinoid and neuroplasticity-related changes as susceptibility factors in a rat model of posttraumatic stress disorder. *Neurobiol Stress*. 2024;32:100662. doi:10.1016/j.ynstr.2024.100662

20. Berg S, Kutra D, Kroeger T, et al. ilastik: interactive machine learning for (bio)image analysis. *Nat Methods*. 2019;16(12):1226-1232. doi:10.1038/s41592-019-0582-9

21. Schneider CA, Rasband WS, Eliceiri KW. NIH Image to ImageJ: 25 years of image analysis. *Nat Methods*. 2012;9(7):671-675. doi:10.1038/nmeth.2089

22. Clarke D, Crombag HS, Hall CN. An open-source pipeline for analysing changes in microglial morphology. *Open Biol*. 2021;11(8):210045. doi:10.1098/rsob.210045

23. Zelena D, Mergl Z, Foldes A, Kovács KJ, Tóth Z, Makara GB. Role of hypothalamic inputs in maintaining pituitary-adrenal responsiveness in repeated restraint. *Am J Physiol Endocrinol Metab*. 2003;285(5):E1110-7. doi:10.1152/ajpendo.00219.2003

24. Toth M, Mikics E, Tulogdi A, Aliczki M, Haller J. Post-weaning social isolation induces abnormal forms of aggression in conjunction with increased glucocorticoid and autonomic stress responses. *Horm Behav*. 2011;60(1):28-36. doi:10.1016/j.yhbeh.2011.02.003

25. Bhavesh R, Lechuga-Vieco A V, Ruiz-Cabello J, Herranz F. T₁-MRI Fluorescent Iron Oxide Nanoparticles by Microwave Assisted Synthesis. *Nanomater (Basel, Switzerland)*. 2015;5(4):1880-1890. doi:10.3390/nano5041880

26. Schwarz AJ, Danckaert A, Reese T, et al. A stereotaxic MRI template set for the rat brain with tissue class distribution maps and co-registered anatomical atlas: application to pharmacological MRI. *Neuroimage*. 2006;32(2):538-550. doi:10.1016/j.neuroimage.2006.04.214

27. Patro R, Duggal G, Love MI, Irizarry RA, Kingsford C. Salmon provides fast and bias-aware quantification of transcript expression. *Nat Methods*. 2017;14(4):417-419. doi:10.1038/nmeth.4197

28. Soneson C, Love MI, Robinson MD. Differential analyses for RNA-seq: Transcript-level estimates improve gene-level inferences. *F1000Research*. 2016;4:1-19. doi:10.12688/F1000RESEARCH.7563.2

29. Love MI, Huber W, Anders S. Moderated estimation of fold change and dispersion for RNA-seq data with DESeq2. *Genome Biol*. 2014;15(12):550. doi:10.1186/s13059-014-0550-8

30. Blighe K, Rana S, Lewis M. EnhancedVolcano: Publication-ready volcano plots with enhanced colouring and labeling.le.

31. Korotkevich G, Sukhov V, Budin N, Shpak B, Artyomov MN, Sergushichev A. Fast gene set enrichment analysis. *bioRxiv*. Published online January 1, 2021:60012. doi:10.1101/060012

32. Wu T, Hu E, Xu S, et al. clusterProfiler 4.0: A universal enrichment tool for interpreting omics data. *Innov (Cambridge*. 2021;2(3):100141. doi:10.1016/j.xinn.2021.100141

33. Varga ZK, Pejtsik D, Tóth M, et al. Improving anxiety research: novel approach to reveal trait anxiety through summary measures of multiple states. *bioRxiv*. Published online January 1, 2024:2023.06.01.543235. doi:10.1101/2023.06.01.543235

34. Wilson CA, Koenig JI. Social interaction and social withdrawal in rodents as readouts for investigating the negative symptoms of schizophrenia. *Eur Neuropsychopharmacol J Eur Coll Neuropsychopharmacol*. 2014;24(5):759-773. doi:10.1016/j.euroneuro.2013.11.008

35. Motz BA, Alberts JR. The validity and utility of geotaxis in young rodents. *Neurotoxicol Teratol*. 2005;27(4):529-533. doi:10.1016/j.ntt.2005.06.005

36. Kaidbey JH, Ranger M, Myers MM, et al. Early Life Maternal Separation and Maternal Behaviour Modulate Acoustic Characteristics of Rat Pup Ultrasonic Vocalizations. *Sci Rep*. 2019;9(1):19012. doi:10.1038/s41598-019-54800-z

37. Biro L, Miskolczi C, Szebik H, et al. Post-weaning social isolation in male mice leads to abnormal aggression and disrupted network organization in the prefrontal cortex: Contribution of parvalbumin interneurons with or without perineuronal nets. *Neurobiol Stress*. 2023;25:100546. doi:10.1016/j.ynstr.2023.100546

38. Papilloud A, Guillot de Suduiraut I, Zanoletti O, Grosse J, Sandi C. Peripubertal stress increases play fighting at adolescence and modulates nucleus accumbens CB1 receptor expression and mitochondrial function in the amygdala. *Transl Psychiatry*. 2018;8(1):156. doi:10.1038/s41398-018-0215-6

39. Choi M, Jang H-S, Son T, et al. Effect Sizes of Cognitive and Locomotive Behavior Tests in the 5XFAD-J Mouse Model of Alzheimer’s Disease. *Int J Mol Sci*. 2023;24(20). doi:10.3390/ijms242015064

40. Tucker LB, Fu AH, McCabe JT. Performance of Male and Female C57BL/6J Mice on Motor and Cognitive Tasks Commonly Used in Pre-Clinical Traumatic Brain Injury Research. *J Neurotrauma*. 2016;33(9):880-894. doi:10.1089/neu.2015.3977

41. Szente L, Aliczki M, Balla GY, et al. Pretrauma cognitive traits predict trauma-induced fear generalization and associated prefrontal functioning in a longitudinal model of posttraumatic stress disorder. *bioRxiv*. Published online January 1, 2024:2024.03.11.584500. doi:10.1101/2024.03.11.584500

42. Gordan ML, Jungwirth B, Ohl F, Kellermann K, Kochs EF, Blobner M. Evaluation of neurobehavioral deficits following different severities of cerebral ischemia in rats: a comparison between the modified hole board test and the Morris water maze test. *Behav Brain Res*. 2012;235(1):7-20. doi:10.1016/j.bbr.2012.07.027

43. Renda CR, Madden GJ. Impulsive choice and pre-exposure to delays: III. Four-month test-retest outcomes in male wistar rats. *Behav Processes*. 2016;126:108-112. doi:10.1016/j.beproc.2016.03.014

44. Serrano NE, Saputra SG, Íbias J, Company M, Nazarian A. Pain-induced impulsivity is sexually dimorphic and mu-opioid receptor sensitive in rats. *Psychopharmacology (Berl)*. 2021;238(12):3447-3462. doi:10.1007/s00213-021-05963-z

45. Bezdjian S, Baker LA, Lozano DI, Raine A. Assessing inattention and impulsivity in children during the Go/NoGo task. *Br J Dev Psychol*. 2009;27(Pt 2):365-383. doi:10.1348/026151008X314919
